# Predicted Metabolic Function of the Gut Microbiota of Drosophila melanogaster

**DOI:** 10.1128/mSystems.01369-20

**Published:** 2021-05-04

**Authors:** Nana Y. D. Ankrah, Brandon E. Barker, Joan Song, Cindy Wu, John G. McMullen, Angela E. Douglas

**Affiliations:** a Department of Entomology, Cornell University, Ithaca, New York, USA; b Center for Advanced Computing, Cornell University, Ithaca, New York, USA; c School of Electrical and Computer Engineering, Cornell University, Ithaca, New York, USA; d Robert Frederick Smith School of Chemical and Biomolecular Engineering, Cornell University, Ithaca, New York, USA; e Department of Molecular Biology and Genetics, Cornell University, Ithaca, New York, USA; KU Leuven

**Keywords:** microbiome, constraint-based modeling, mutualism, competition, cross-feeding, *Drosophila*

## Abstract

An important goal for many nutrition-based microbiome studies is to identify the metabolic function of microbes in complex microbial communities and their impact on host physiology. This research can be confounded by poorly understood effects of community composition and host diet on the metabolic traits of individual taxa. Here, we investigated these multiway interactions by constructing and analyzing metabolic models comprising every combination of five bacterial members of the *Drosophila* gut microbiome (from single taxa to the five-member community of *Acetobacter* and *Lactobacillus* species) under three nutrient regimes. We show that the metabolic function of *Drosophila* gut bacteria is dynamic, influenced by community composition, and responsive to dietary modulation. Furthermore, we show that ecological interactions such as competition and mutualism identified from the growth patterns of gut bacteria are underlain by a diversity of metabolic interactions, and show that the bacteria tend to compete for amino acids and B vitamins more frequently than for carbon sources. Our results reveal that, in addition to fermentation products such as acetate, intermediates of the tricarboxylic acid (TCA) cycle, including 2-oxoglutarate and succinate, are produced at high flux and cross-fed between bacterial taxa, suggesting important roles for TCA cycle intermediates in modulating *Drosophila* gut microbe interactions and the potential to influence host traits. These metabolic models provide specific predictions of the patterns of ecological and metabolic interactions among gut bacteria under different nutrient regimes, with potentially important consequences for overall community metabolic function and nutritional interactions with the host.

**IMPORTANCE** *Drosophila* is an important model for microbiome research partly because of the low complexity of its mostly culturable gut microbiota. Our current understanding of how *Drosophila* interacts with its gut microbes and how these interactions influence host traits derives almost entirely from empirical studies that focus on individual microbial taxa or classes of metabolites. These studies have failed to capture fully the complexity of metabolic interactions that occur between host and microbe. To overcome this limitation, we reconstructed and analyzed 31 metabolic models for every combination of the five principal bacterial taxa in the gut microbiome of *Drosophila*. This revealed that metabolic interactions between *Drosophila* gut bacterial taxa are highly dynamic and influenced by cooccurring bacteria and nutrient availability. Our results generate testable hypotheses about among-microbe ecological interactions in the *Drosophila* gut and the diversity of metabolites available to influence host traits.

## INTRODUCTION

Microbiomes associated with animals are variable in taxonomic composition and impact on host traits (1–6). This has generated two linked challenges: to understand the causes of the variability and to predict how the microbiome may interact with other variables, such as host genotype, age, and activity, as well as diet, to determine host traits (7–11). Strategies to reduce and manage these highly complex interactions include the use of simplified microbial communities of defined taxonomic composition (12–14), along with modeling to investigate patterns across larger scales of parameter space than is technically feasible by empirical study (15–19).

The gut microbiota of *Drosophila* provides an attractive system to study taxonomically simple microbiomes because its microbiome is naturally of low diversity, generally with fewer than 20 bacterial species in laboratory culture, and microbiomes of defined composition can readily be generated and maintained (20, 21). In most laboratory cultures, the microbiome is dominated by acetic acid bacteria (AABs), generally of the genus *Acetobacter* (*Alphaproteobacteria*), and lactic acid bacteria (LABs) of the genus *Lactobacillus* (*Firmicutes*) (20), although some studies have reported strong representation of other taxa, including *Gammaproteobacteria* (e.g., *Stenotrophomonas* spp.) and lactobacilli (e.g., *Enterococcus* spp.) (22, 23). Studies on associations with a single bacterial isolate and with communities of 2 to 5 microbial taxa have demonstrated that both individual taxa and communities can, variously, contribute to B vitamin nutrition, reduce lipid content, influence development rates, life span, and fecundity, and modulate olfactory and egg-laying behavior (14, 24–30). In several studies, these microbiome effects on host traits have been shown to vary with diet composition, and they have been linked to among-microbe interactions that influence both the abundance and metabolic activity of individual microbial taxa (14, 25, 29–31). However, the relationship between diet, community composition, and metabolic function of the microbiome remains poorly understood.

The goal of this study was two-fold: first, to determine the combined effects of community composition and nutrient availability on the metabolic interactions among members of the *Drosophila* gut microbiota; and second, to establish how these interactions shape the abundance of the microorganisms in the community and the production of metabolites that may influence host traits. We adopted a modeling approach, specifically to construct and analyze the metabolic model for every combination of 5 bacterial taxa isolated from the *Drosophila* gut microbiome, including Acetobacter fabarum, Acetobacter pomorum, Acetobacter tropicalis, Lactobacillus brevis, and Lactobacillus plantarum. This choice of taxa enabled us to examine interactions among species of different taxonomic relatedness and metabolic function. Within the *Acetobacteraceae*, the closely related *A. fabarum* and A. pomorum are assigned to a different subgroup of *Acetobacter* from *A. tropicalis* (32), and the homofermentative *L. plantarum* and heterofermentative L. brevis are members of different phylogenetic groups of *Lactobacillus* (33). Members of the genus *Acetobacter* and *Lactobacillus* are well represented across both field and lab *Drosophila* populations (34–37), and have been widely used to investigate the impact of gut microbes on host physiology (14, 30, 38, 39). The metabolic network for each species was reconstructed from annotated metabolism genes in the sequenced genome, and the networks for the different species in each community integrated into community models. We then applied the SteadyCom framework (40) to quantify the steady-state composition of each community and to predict the metabolic flux within and between the bacteria contributing to each community. Although a number of community modeling approaches are currently available (16, 41–45), we used SteadyCom because other community modeling approaches generally assume fixed community composition and lack constraints that prevent fast-growing organisms from displacing other microbes in the community regardless of nutrient availability in the environment. SteadyCom applies more ecologically relevant constraints by imposing a single, time-averaged constant growth rate across all members of a community to ensure coexistence and stability as predicted to occur in animal guts. SteadyCom is also applicable to established constraint-based modeling approaches, such as flux variability analysis, an important tool for determining the robustness of metabolic models in various simulation conditions. This approach enabled us to assess how community composition is influenced by antagonistic and mutualistic metabolic interactions, and to evaluate how among-microbe interactions can dictate overall metabolic outputs from the community.

## RESULTS

### Growth of *Drosophila* gut bacterial communities under different nutrient regimes.

**FIG 1:**
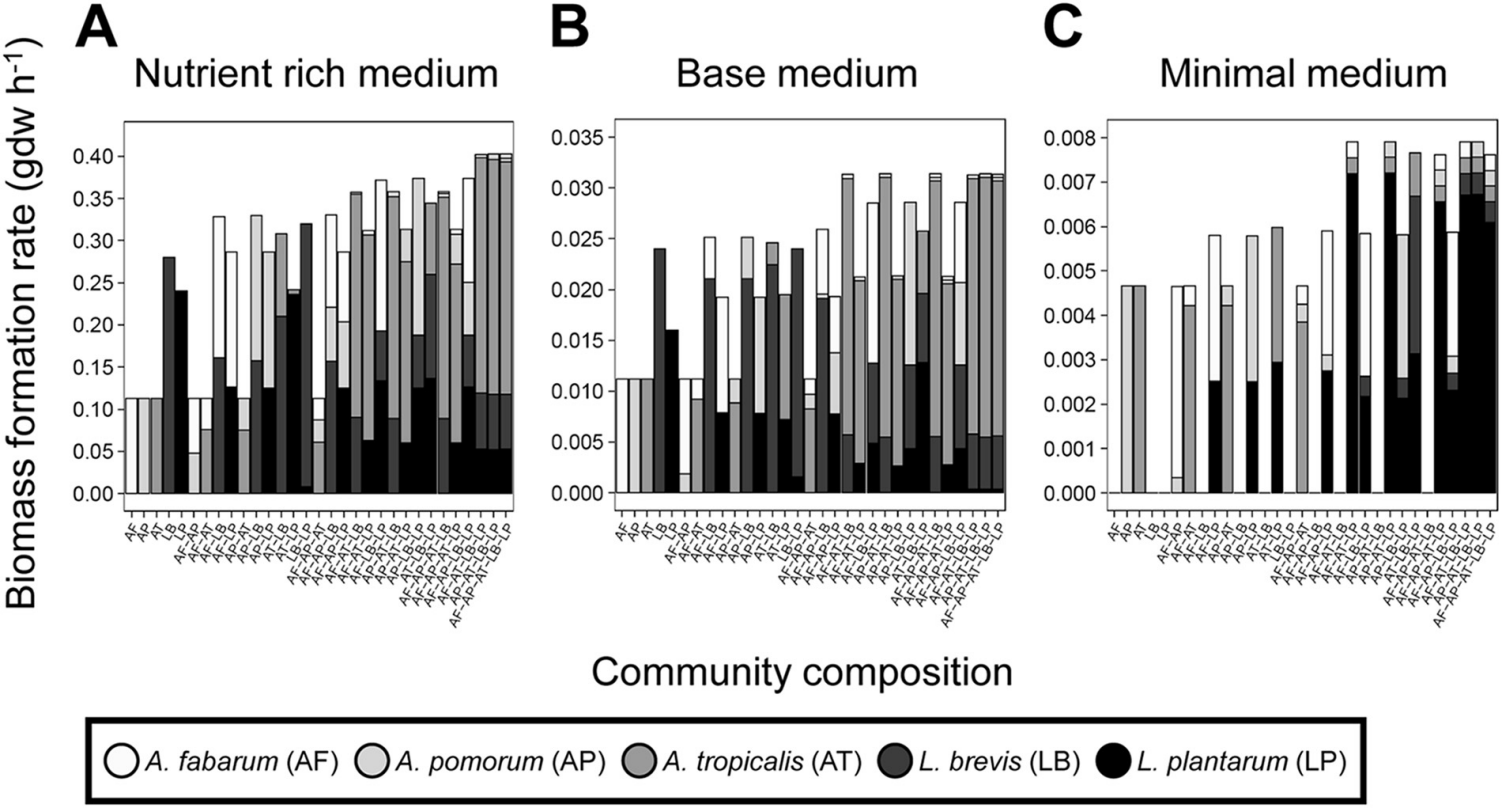
Bacterial growth dynamics on media of different nutrient content. Growth dynamics displayed as biomass formation rate predicted for growth nutrient-rich medium (A), base medium (B), and minimal medium (C).

Our first analysis tested for growth *in silico* of the 31 possible communities of the five test bacteria under three nutrient regimes (Fig. 1). As predicted, all five single-species communities displayed growth in both the base medium, comprising the complete set of nutrients required for growth by all the bacteria, and the nutrient-rich medium, in which the complete set of nutrients was provided in excess. However, only two bacteria, the acetic acid bacteria (AAB) A. pomorum and *A. tropicalis*, grew on the minimal medium containing glucose, glycerol, ammonia, sulfate, and phosphate as primary sources of carbon, nitrogen, sulfur, and phosphorus, respectively. Growth of the third AAB, *A. fabarum*, was rescued by coculture with any other AAB or with *L. plantarum*, one of the two lactic acid bacteria (LAB), but not with L. brevis. Similarly, growth of *L. plantarum* was rescued by coculture with any AAB but not with L. brevis. The coculture requirements of L. brevis were greater, requiring both *L. plantarum* and at least one AAB. The failure of *A. fabarum*, *L. plantarum*, and L. brevis to grow *in silico* on the minimal medium was a consequence of their auxotrophy for amino acids such as arginine and the peptidoglycan precursor diaminoheptanedioate. These metabolites are released from bacterial species, e.g., A. pomorum and *A. tropicalis*, so as to rescue growth.

**FIG 2:**
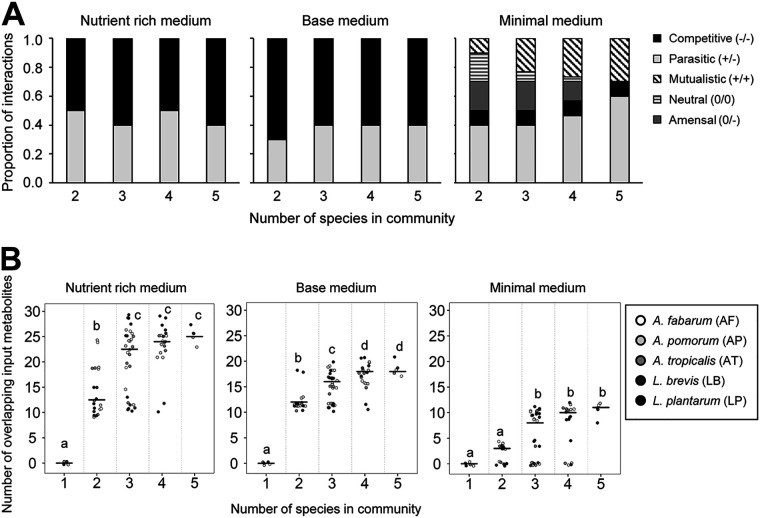
Ecological interactions in simulated communities of different diversity. (A) Impact of coculture and medium on the sign of interactions (+, beneficial; −, antagonistic; 0, neutral) between bacteria in the three test media. No commensal interactions were observed in any media type. (B) Overlapping metabolites consumed by bacteria in the 31 simulated communities. Significantly different (*P* < 0.05) groups by Tukey’s HSD *post hoc* test are indicated by different letters.

Further inspection of the data in Fig. 1 revealed the diverse effects of coculture on growth dynamics of individual bacteria. For example, coculture with *A. tropicalis* tended to reduce the growth of the other bacteria on the nutrient-rich medium, while the growth of AABs on the minimal medium was largely unaffected by coculture with *L. plantarum* in two-member communities, but strongly repressed in communities of two or three AABs with *L. plantarum* (Table S1 in the supplemental material). To investigate these effects systematically, we classified coculture interactions as sets of binary interactions: (i) competitive if both organisms displayed reduced growth in coculture; (ii) parasitic if the growth of one member was enhanced at the expense of another; (iii) mutualistic if both organisms displayed increased growth in coculture; (iv) commensal if one organism displayed increased growth with no change in the other; (v) amensal if one organism displayed reduced growth with no change in the other; and (vi) neutral if the growth of both organisms was unaltered. Increases and decreases in growth were determined by comparing the growth of a microbe in isolation to its growth in coculture. In the nutrient-rich and basal media, the interactions were exclusively antagonistic: 50 to 70% of the interactions were competitive, and the remainder were parasitic (Fig. 2A, Table S1). In the minimal medium, parasitic interactions predominated, but competitive, mutualistic, amensal, and neutral interactions also occurred (Fig. 2A). Notably, mutualistic interactions accounted for 12% of the interactions in two-member communities and increased progressively to 30% of the interactions in the five-member community. A large proportion (∼80%) of neutral interactions occur between *A. fabarum* and L. brevis and these interactions switch to mutualistic interactions when *L. plantarum* is added to the community (Table S1C). Similarly, ∼66% of amensal interactions occur between L. brevis and at least one AAB (Table S1C). L. brevis-AAB amensal interactions switch to parasitic (L. brevis growth increases, AAB growth decreases) in more complex communities with the addition of *L. plantarum*. The switch in interaction type is facilitated by *L. plantarum* production of meso-2,6-diaminoheptanedioate, an essential metabolite for L. brevis growth.

10.1128/mSystems.01369-20.1 TABLE S1 Predicted changes to bacterial growth (gdw h-1) in coculture compared to growth in monoculture rich medium (A), base medium (B), or minimal medium (C). Download Table S1, PDF file, 0.2 MB. Copyright © 2021 Ankrah et al. 2021 Ankrah et al. https://creativecommons.org/licenses/by/4.0/ This content is distributed under the terms of the Creative Commons Attribution 4.0 International license.

### Patterns of metabolite consumption and release.

We hypothesized that the competitive interactions in the simulated bacterial communities (Fig. 2A) were underlain by the coconsumption of individual nutrients by two or more bacteria in the community; these nutrients may be constituents of the medium or, in communities with three or more members, derived from other bacteria. Additionally, we hypothesized that parasitic interactions involved the unidirectional cross-feeding of metabolites from the bacterium displaying depressed growth to the bacterium displaying increased growth in coculture, while mutualistic interactions involved the reciprocal transfer of metabolites that were synthesized and released by one bacterium and required for growth by the other bacterium.

For our first analysis of the incidence of competition, we quantified the number of individual metabolites consumed by more than one bacterium in the simulated communities (Fig. 2B, Table S2A to C, Dataset 1). As shown in Fig. 2B, the number of nutrients shared between two bacteria increased with both community complexity and nutrient content of the growth medium. The number of shared metabolites increased significantly between two-member and three-member communities, however, increases between three- to four-member and four- to five-member communities were mostly not significant for all media types (Table S2D). Our observation of strong pairwise interactions within communities of low complexity (<4 members) is similar to data from vertebrate systems showing how pairwise interactions between gut microbial communities weaken as communities increase in complexity (46). Consistent with the relatively low incidence of competitive interactions in the minimal medium, the greatest number of overlapping input metabolites recorded for any interaction in this medium was 12, which was half or less of the equivalent values, 21 and 29 for the basal medium and nutrient-rich medium, respectively (Fig. 2B).

10.1128/mSystems.01369-20.2 TABLE S2 Predicted number of inputs and outputs from bacteria in rich medium (A), base medium (B), or minimal medium (C). (D) Summary statistics for Fig. 2B. Download Table S2, PDF file, 0.2 MB. Copyright © 2021 Ankrah et al. 2021 Ankrah et al. https://creativecommons.org/licenses/by/4.0/ This content is distributed under the terms of the Creative Commons Attribution 4.0 International license.

**FIG 3:**
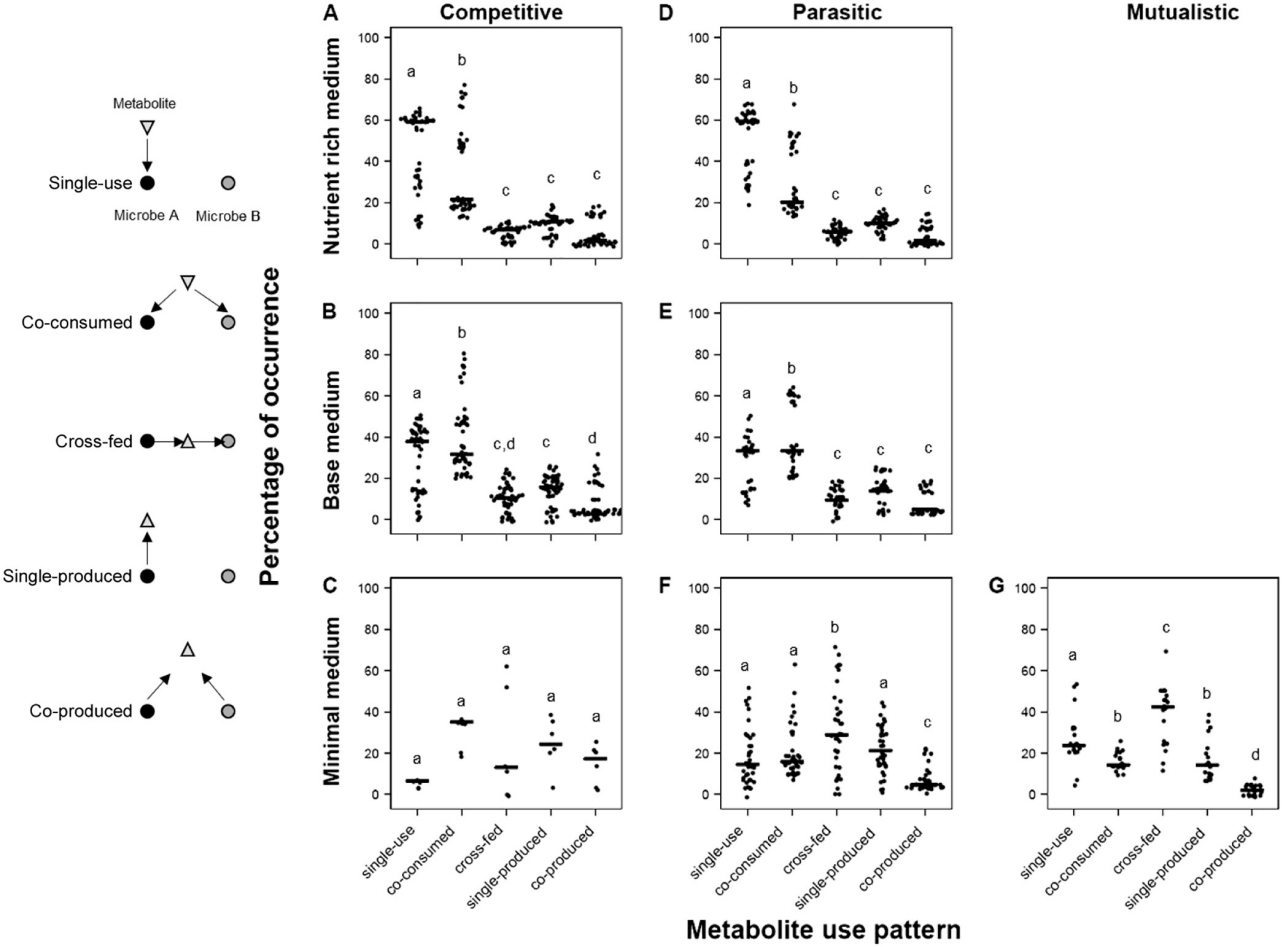
Metabolite use patterns associated with competitive, parasitic, and mutualistic growth outcomes. Each point represents the frequency of a metabolite use pattern associated between all pairwise interactions across all communities from 2 to 5 members. The relative frequency of metabolite use is calculated by dividing the number of times a metabolite is used in a particular pattern (single-use, co-consumed, cross-fed, single-produced, or coproduced) by the total number of times the metabolite is produced or consumed in the 31 simulated communities. Black bars indicate the median frequency of occurrence for each metabolite use pattern. Significantly different (*P* < 0.05) groups by Tukey’s HSD *post hoc* test are indicated by different letters. The panel at the left summarizes the five different types of metabolite use (triangle, metabolite; open and closed circles, co-occurring Microbe A and Microbe B, respectively).

To investigate the specific metabolic drivers of the antagonistic growth interactions, i.e., both competition and parasitism, we determined the input and output metabolites of each bacterium in every community. A total of 100 unique metabolites was predicted to be produced or consumed. We classified each metabolite by the frequency of its consumption by members of the gut community (Fig. 3, Table S3A to C). Our data show distinct metabolite use patterns associated with competitive, parasitic, and mutualistic growth outcomes. Competitive growth interactions were significantly dominated by single-use or coconsumption in the rich and base media, but not in the minimal medium (Fig. 3A to C, Table S3D). Similarly, parasitic interactions were significantly dominated by single-use or coconsumption in rich and base media, and single-produced, and cross-fed metabolite use patterns dominated minimal medium parasitic interactions (Fig. 3D to F, Table S3D). Mutualistic growth interactions, observed only in the minimal medium, were characterized by the dominance of cross-feeding interactions, which made up a significantly larger proportion of all interaction types (Fig. 3G, Table S3D).

10.1128/mSystems.01369-20.3 TABLE S3 Predicted community metabolite use patterns for competitive, parasitic and mutualistic interactions in rich medium (A), base medium (B), or minimal medium (C). (D) Summary statistics for Fig. 3. Download Table S3, PDF file, 0.2 MB. Copyright © 2021 Ankrah et al. 2021 Ankrah et al. https://creativecommons.org/licenses/by/4.0/ This content is distributed under the terms of the Creative Commons Attribution 4.0 International license.

Our data also show shifts in metabolite use profiles with depletion of nutrients in the growth medium. In the nutrient-rich medium, single-use consumption of metabolites predominated, while metabolites in the base and minimal media had more diverse metabolite use profiles, with an increasing representation of cross-fed, single-produced, and coproduced metabolites (Fig. 3).

**FIG 4:**
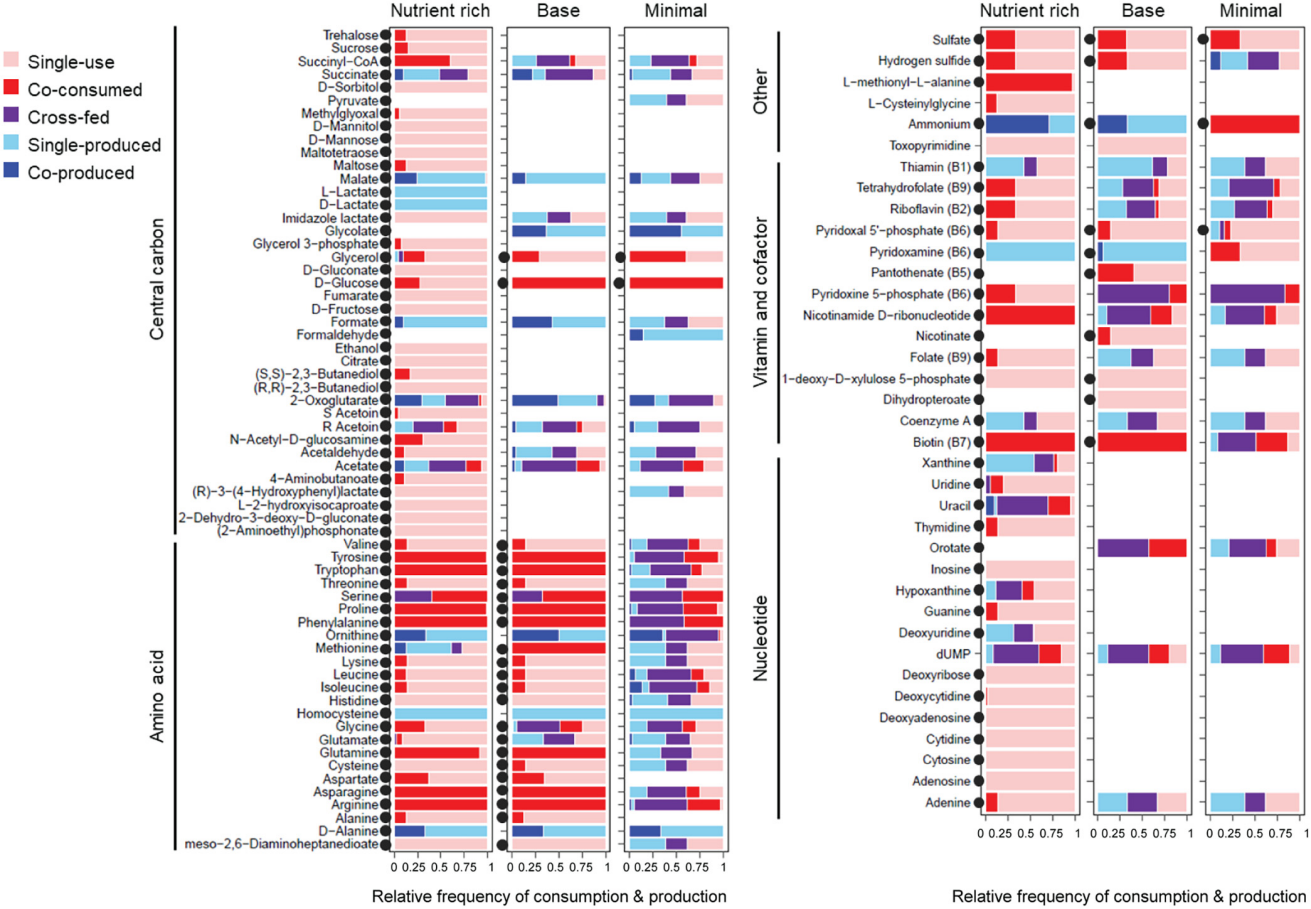
Metabolite use patterns for metabolite classes of amino acids, carbon, nucleotides, vitamins, and cofactors. Tick marks on the *x* axis indicate the relative frequency of the consumption or production of a metabolite and range from 0 to 1 at 0.25 increments. The relative frequency of metabolite use is calculated by dividing the number of times a metabolite is used in a particular pattern (single-use, co-consumed, cross-fed, single-produced, or coproduced) by the total number of times the metabolite is produced or consumed in the 31 simulated communities. Black circles demarcate metabolites that are initially present in each medium.

We then investigated the identity of metabolites in the different metabolite use patterns. Metabolite groups with the highest number of co-consumed metabolites were amino acids and B vitamins (Fig. 4, Table S4A to C). Tyrosine, tryptophan, proline, phenylalanine, glutamine, asparagine, and arginine were the most frequently co-consumed amino acids, and biotin (B7) was the most co-consumed B vitamin. However, some B vitamins and cofactors were cross-fed, notably thiamine (B1), pyridoxine 5-phosphate (B6), nicotinamide d-ribonucleotide, riboflavin (B2), tetrahydrofolate (B9), and coenzyme A (Fig. 4). Among the carbon compounds, only glucose and glycerol were consistently co-consumed in all three media. Other carbon compounds displayed more diverse use profiles that differed for each growth medium. For instance, malate and formate were exclusively produced in the nutrient-rich and base media and consumed or cross-fed in the minimal medium (Fig. 4), while lactate and 2,3 butanediol were exclusively produced in the nutrient-rich medium. Intermediates of the TCA cycle, including 2-oxoglutarate, succinate, succinyl-CoA, and fermentation products acetate, acetaldehyde, and acetoin were cross-fed carbon at high frequency, while serine, glutamate, and glycine were the most cross-fed amino acids (Fig. 4). Among the nucleotides, the pyrimidine deoxyuridine 5′-phosphate (dUMP) simultaneously ranked as the most co-consumed and cross-fed nucleotide (Fig. 4).

10.1128/mSystems.01369-20.4 TABLE S4 Metabolite use pattern in rich medium (A), base medium (B), or minimal medium (C). (D) Effect of community size and medium type on metabolite richness. Tests with significant *P* values are shown in bold. Download Table S4, PDF file, 0.3 MB. Copyright © 2021 Ankrah et al. 2021 Ankrah et al. https://creativecommons.org/licenses/by/4.0/ This content is distributed under the terms of the Creative Commons Attribution 4.0 International license.

### Metabolic roles of individual bacteria.

**FIG 5:**
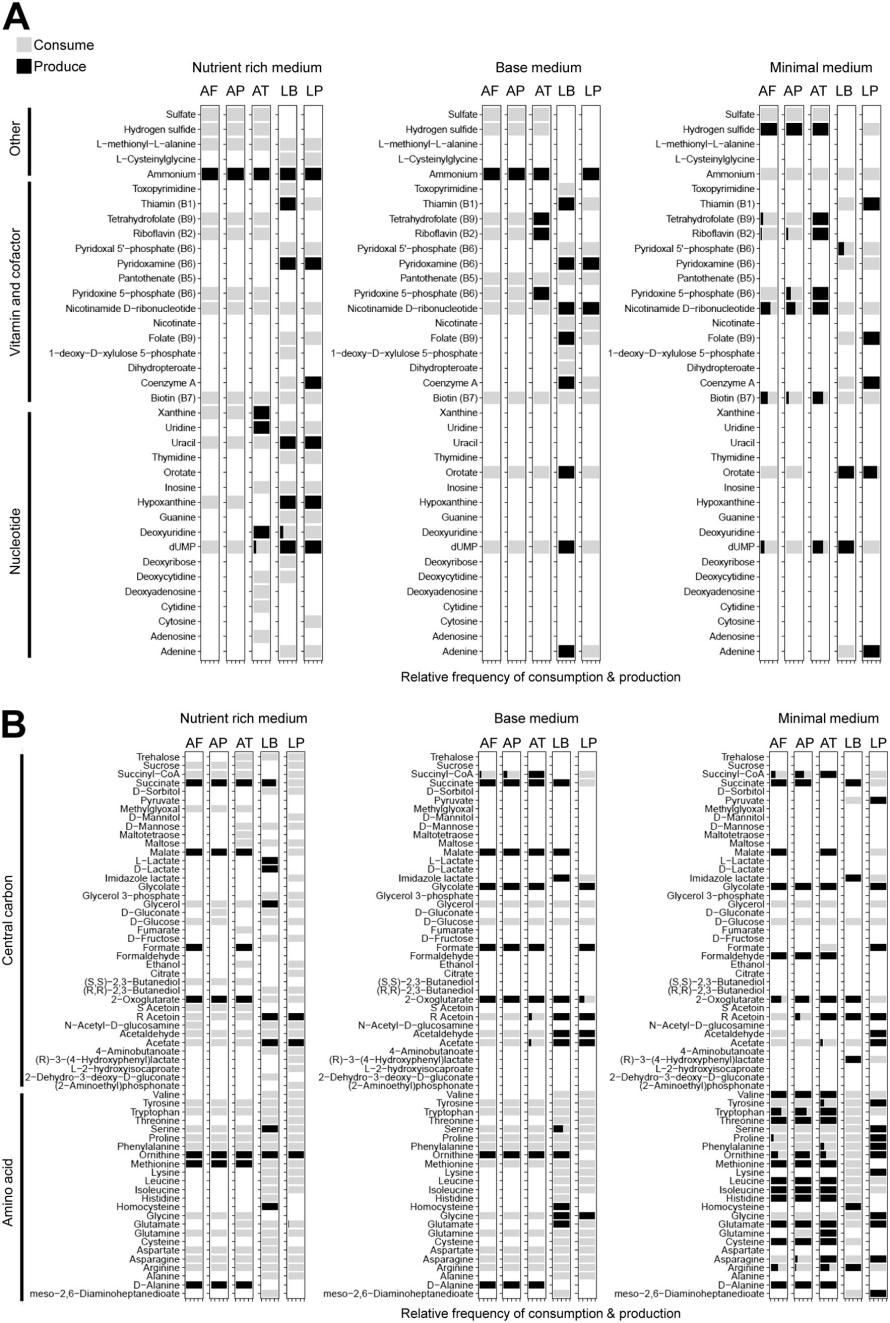
Metabolic roles of individual bacteria. Predicted metabolite production and consumption profiles for metabolite classes nucleotides, vitamins, and cofactors (A) and amino acid and carbon (B). The two-letter abbreviations at the top of each plot represent individual bacteria: AF, Acetobacter fabarum; AP, Acetobacter pomorum; AT, Acetobacter tropicalis; LB, Lactobacillus brevis; LP, Lactobacillus plantarum. Tick marks on the *x* axis indicate the relative frequency of the consumption or production of a metabolite and range from 0 to 1 at 0.25 increments.

Our next analyses focused on metabolite production and consumption profiles of individual gut bacteria. Our simulations show that the metabolic role of individual gut bacteria as source or sink varies with the identity of coculture microbe and across the three media for many metabolites, but is generally conserved across communities within the same medium type (Fig. 5, Table S4D). For instance, all five gut bacteria produced ammonia in the nutrient-rich and base media but consumed ammonia in the minimal medium (Fig. 5A). In base and rich media, most microbes, except *A. tropicalis*, displayed committed roles for the production or consumption of all metabolites (Table S5A to C). *A. tropicalis* alternated roles as a producer and consumer for up to 6% of all metabolites (Table S5A to C). In the minimal medium, all three acetic acid bacteria displayed variable roles as producers and consumers for up to ∼30% of all metabolites (Table S5A to C). As an example, representatives of *Acetobacter* alternated as sources and sinks for acetate, arginine, ornithine, and succinyl-CoA in the minimal medium, depending on the number and identity of cooccurring bacteria (Fig. 5B).

10.1128/mSystems.01369-20.5 TABLE S5 Predicted total number of times metabolite is consumed or produced by individual bacteria in all simulations in rich medium (A), base medium (B), or minimal medium (C). (D) Effect of taxa, community size, and medium type on metabolite consumption and release rates. Tests with significant *P* values are shown in bold. Download Table S5, PDF file, 0.3 MB. Copyright © 2021 Ankrah et al. 2021 Ankrah et al. https://creativecommons.org/licenses/by/4.0/ This content is distributed under the terms of the Creative Commons Attribution 4.0 International license.

Furthermore, the bacterial taxa had distinctive metabolic characteristics. All three *Acetobacter* species were sinks for glycine, serine, proline, acetaldehyde, and methylglyoxal and producers of d-alanine, cysteine, histidine, isoleucine, leucine, valine, formaldehyde, malate, and succinate (Fig. 5A), and *A. tropicalis* was, additionally, an important source of B vitamins, including tetrahydrofolate (B9), riboflavin (B2), pyridoxine 5-phosphate (B6), and biotin (B7) (Fig. 5B). Both *Lactobacillus* species produced acetoin and were sinks for tryptophan and the branched-chain amino acids isoleucine, leucine, and valine. L. brevis was consistently a source of succinate and deoxyuridine 5′-phosphate (dUMP) and a sink for most amino acids and meso-2,6-diaminoheptanedioate (required for peptidoglycan synthesis). *L. plantarum* was a sink for arginine and succinate and a source of pyruvate and meso-2,6-diaminoheptanedioate.

### Effect of community size and taxa on metabolite richness.

**FIG 6:**
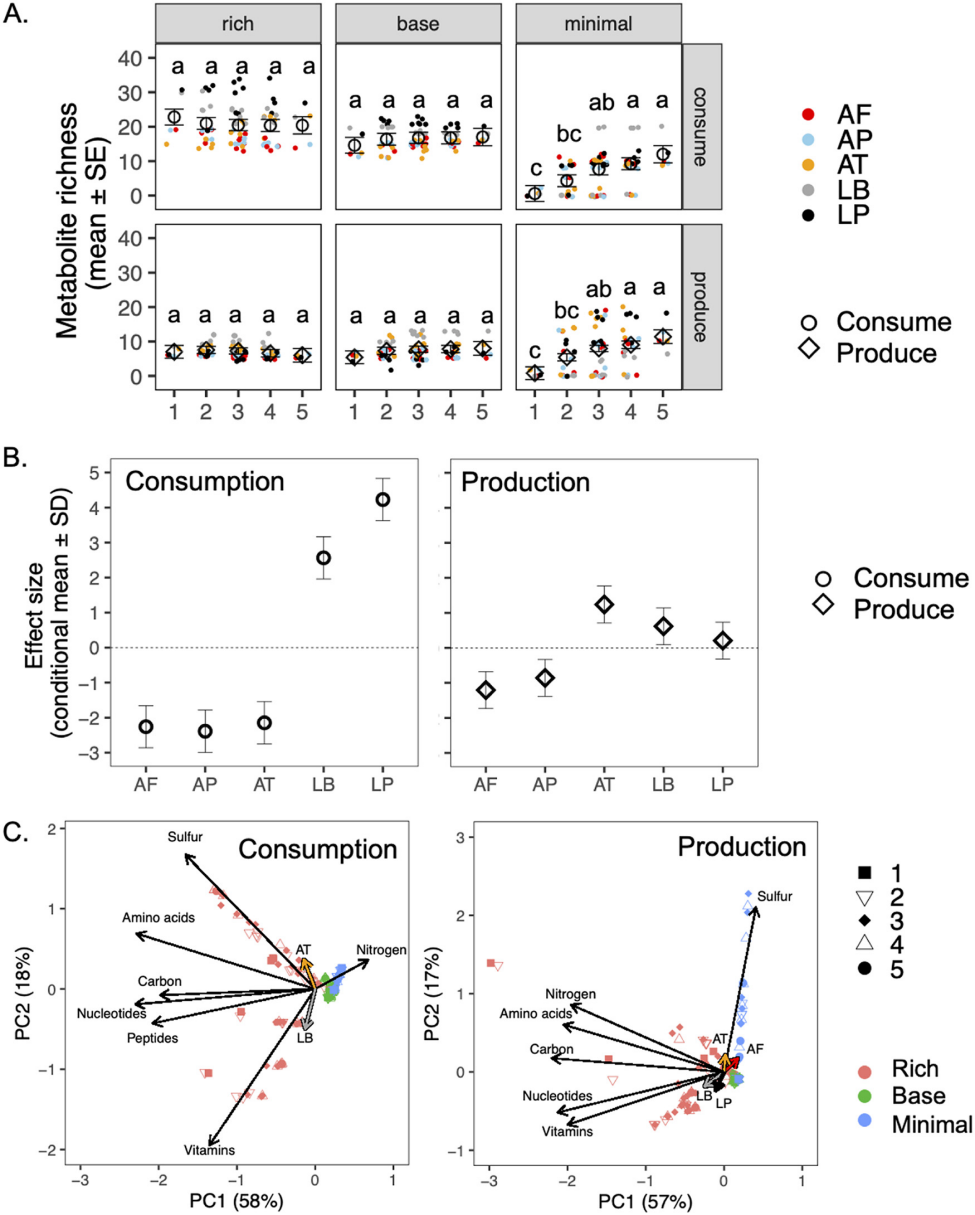
Metabolic function of simulated microbial taxa under different conditions. (A) Effect of community size on metabolite richness. Metabolite richness is calculated as the number of metabolites either consumed or released by a given taxon in each microbial treatment and medium combination. Effect of community size for each medium is indicated with the estimated marginal mean (open circles or diamonds) and standard error (SE) from ANOVA models. Letters indicate results from *post hoc* Tukey’s test, which was conducted separately for each medium. Closed, colored circles indicate individual metabolite richness values for each taxon under each condition. Effect test results are displayed in Table S4D in the supplemental material. (B) Global effect of species identity on metabolite richness for the number of compounds consumed and released. The conditional mean and standard deviation are displayed for the best linear unbiased prediction. Dotted lines indicate the grand mean for metabolite richness across all species. (C) Principal-component analysis (PCA) correlating metabolite consumption or release rates with community size, medium type, and microbial presence. Black arrows indicate metabolite type scores, and colored arrows display the correlation vectors for microbial presence (only significant vectors are plotted). The percent variance explained by each axis is shown in parentheses.

We next considered how the number of metabolites consumed or released by individual bacteria (i.e., metabolite richness) was influenced by the number and identity of other bacteria in the community. For the nutrient-rich and basal media, the number of taxa in a community did not significantly influence the number of metabolites consumed or released by individual taxa (Fig. 6A, Table S4D). However, on the minimal medium, as the number of taxa in a community increased, the number of metabolites consumed and released also increased (Fig. 6A).

The metabolic function of individual microbial taxa significantly varied across all media types (Table S4D). In particular, both *Lactobacillus* species consumed more metabolites than the three *Acetobacter* species. *A. tropicalis* released more metabolites on average than the other two *Acetobacter* species, and both *Lactobacillus* species released slightly more metabolites on average than the grand mean (Fig. 6B).

The types of metabolites consumed or released varied with the identity and number of species in the community (Fig. 6C, Table S5D). *A. tropicalis* and L. brevis had the strongest effect on consumption rates, especially in the nutrient-rich medium; *A. tropicalis* positively correlated with consumption of sulfur-containing metabolites, while L. brevis increased with vitamin consumption (Fig. 6C). For metabolite release, *Lactobacillus* species had the greatest impact in the nutrient-rich medium, correlated with vitamin and nucleotide release, while the *Acetobacter* species were more important in the minimal depleted medium and *A. tropicalis*, in particular, was strongly correlated with the release of sulfur-containing metabolites (Fig. 6C). A. pomorum consistently had no effect on consumption or production rates (Table S5D).

### Net outputs from the bacterial communities.

Our final analysis addressed the metabolic products of the 31 bacterial communities in each of the three media. These products are candidate bacterial-derived metabolites that are available to the host animal. A total of 21 metabolites was predicted to be made available to the host (Table S6, Dataset 2), 19 of which were produced in the nutrient-rich medium, 12 in the base medium, and 5 in the minimal medium. Some of the 21 metabolites have been previously demonstrated to play important roles in *Drosophila* physiology. For instance, acetate (26, 47, 48) and succinate (49) reduce host TAG levels, and microbe-derived amino acids rescue *Drosophila* growth on amino acid-deficient diets (30, 50) and double *Drosophila* life span on low-protein diets (51).

10.1128/mSystems.01369-20.6 TABLE S6 Metabolites predicted to be available to the host. Download Table S6, PDF file, 0.1 MB. Copyright © 2021 Ankrah et al. 2021 Ankrah et al. https://creativecommons.org/licenses/by/4.0/ This content is distributed under the terms of the Creative Commons Attribution 4.0 International license.

Three metabolites (acetate, d-alanine, and homocysteine) were predicted to be made available to the host under all three diet conditions. In addition, the central carbon metabolites predicted to be released from bacteria in at least one medium include 2-oxoglutarate, formaldehyde, formate, glycolate, lactate, malate, and succinate.

## DISCUSSION

Our *in silico* study of metabolic interactions among *Drosophila* gut bacteria yielded two key findings. First, the pattern of metabolite consumption and release by individual bacteria and communities is dynamic, varying with nutrient conditions and community composition. Second, ecological interactions identified from the growth patterns of the bacteria (competition, mutualism, etc.) are underpinned by a diversity of metabolic interactions, with evidence that the bacteria tend to compete for certain classes of nutrients (e.g., amino acids and B vitamins) more frequently than for others, particularly carbon sources.

Our modeling is based on several simplifying assumptions. In particular, we assume that the different bacterial species are in close proximity, such that among-species flux of metabolites is unimpeded. Although there is very limited information on the spatial organization of gut microorganisms in *Drosophila*, bacteria in other hosts can be planktonic in the gut lumen or adhere to the gut wall, often as single-species or structured multispecies colonies (52–55), and these different spatial patterns both affect and are influenced by abiotic conditions, nutrient availability, metabolite exchange, and types of growth interactions between species (56–58). For example, commensal and neutral interactions, which are likely to occur among spatially isolated microbes, are not captured in most of our simulations. Furthermore, our models address primary metabolism exclusively and are not designed to investigate the effects of secondary metabolite-mediated interactions, e.g., interference competition among bacteria mediated by toxins (59, 60) or differential susceptibility of bacteria to host immune factors (61–64). Despite these limitations, many of our model outputs are consistent with published empirical data on the metabolic function of *Drosophila* gut bacteria, particularly the production by individual bacterial species of specific fermentation products (24, 65), amino acids (51, 66), and B vitamins (29, 39, 67). Furthermore, the predicted incidence of different ecological interactions, as deduced from biomass production in the various communities, agrees largely with published data reporting a predominance of antagonistic interactions among *Drosophila*-associated gut microbes (14) and other microbial communities (60, 68–70). Our observation that nutrient-poor conditions favor mutualistic interactions, especially in more complex communities, is also consistent with both predictions and empirical data for other microbial systems, e.g., references 71–73. Taken together, these considerations indicate that our modeling approach is robust. It can be used with confidence to investigate patterns in the metabolic consequences of various nutrient availability and community composition over a larger range of conditions than is technically realistic for empirical study.

Genome-scale metabolic modeling, as used here, brings into sharp focus the complexity of metabolic interactions among microorganisms. This provides a different perspective from empirical studies that, generally, focus on a single class of nutrients, e.g., short-chain fatty acids or B vitamins. In particular, the metabolic traits of an individual bacterium are not fixed but strongly influenced by the nutrient environment and the presence and identity of cooccurring microorganisms. For example, our models predict that the *Lactobacillus* species are net producers of uracil only under nutrient-rich conditions (Fig. 5). Bacterial-derived uracil has been shown to induce a proinflammatory state in the *Drosophila* gut via DUOX-mediated production of reactive oxygen species (74), and our data raise the possibility that the effect of *Lactobacillus* on the immunological status of the gut may be influenced by dietary factors. Similarly, the finding that the net production of several B vitamins by *Acetobacter* varies with the presence and identity of cooccurring bacteria (Fig. 5A) suggests that studies exclusively using associations with single bacterial taxa may not capture the full complexity of B vitamin provisioning by the *Drosophila* gut microbiome.

The complexity of the metabolic interactions among the bacterial species also influences the sign of ecological interactions. Various empirical analyses have demonstrated how an ecological interaction can be driven by a single metabolic interaction, e.g., competition for a single resource (75–77) or mutualism by reciprocal cross-feeding of a pair of metabolites, each produced by one microorganism and required by the other (78, 79). However, as summarized in Fig. 3, the totality of the metabolic relationship between interacting microorganisms includes multiple classes of metabolic interaction. A relatively minor change in the uptake/release of metabolite(s), in response to a change in nutrient availability or community composition, could result in the transition to a different ecological relationship. For example, the switch from a competitive interaction to parasitism may contribute to the bloom of a microorganism previously held in check by competition (80).

Further elaboration of metabolic models, as used here and, for example, by reference 70, offers the opportunity to investigate how subtle changes in metabolite flux in communities of different complexity and different nutrient regimes can lead to a switch between different ecological states of the microbiome and its interaction with the host.

The complexity and variation in metabolic interactions among the gut bacteria are, however, overlain by several broad patterns with respect to both metabolite class and bacterial species. Considering metabolites first, a key output of this study is that the pattern of metabolite use differed between substitutable and nonsubstitutable nutrient sources, i.e., where other resources can be utilized as an equivalent resource versus where no equivalent resource is available (81). Consistent with the generality that organisms tend to compete less for substitutable than for nonsubstitutable resources (82), the *Drosophila* gut bacteria competed more for nonsubstitutable nutrients, such as amino acids and B vitamins, than substitutable nutrients, such as intermediates in central carbon metabolism. These patterns have implications for the health and wellbeing of the host. In particular, microbial consumption of dietary nutrients can deplete nutrient availability to the host (83), and among-microbe competition is predicted to alter host access to both critical nutrients and metabolites that influence the signaling pathways in *Drosophila* (26, 27) and other animals (84, 85).

The five bacteria selected for this analysis varied in their genetic capacity for metabolic function, particularly between the *Acetobacter* and *Lactobacillus* species (Fig. 6, see also reference 38). Nevertheless, our modeling revealed substantial functional redundancy in metabolite production among the different bacteria; except for meso-2,6-diaminoheptanedioate, produced by *L. plantarum*, we identified no metabolites produced exclusively by a single species. This metabolic redundancy is consistent with the evidence that taxonomically different microbial communities can be functionally equivalent in *Drosophila* (86) and other animals (4, 87). The unique metabolic function of *L. plantarum* highlights the role of this bacterium in microbial community interactions, including its potential as a probiotic. The beneficial effects of *L. plantarum* in the *Drosophila* system have been attributed to its capacity to promote protein assimilation from the diet (27). Our observation that *L. plantarum* provisions cell wall constituents, B vitamins, and amino acids for auxotrophic bacteria in the gut provides additional metabolic routes by which *L. plantarum* may promote the overall diversity of the gut microbiota.

In conclusion, this study demonstrates how *in silico* approaches can yield mechanistic insight into the metabolic traits of individual microbes and communities, and how these traits can influence metabolite levels that impact host physiology. Our models identify patterns by which microbial communities interact and respond to changes in nutrient input from the host and allow the generation of testable hypotheses for more targeted empirical studies. Substantial insight into how variations in microbiomes impact host health and metabolism have been gained from combining metabolic modeling with empirical studies (88, 89) and the simplicity of the *Drosophila* system presents an ideal model system to combine *in silico* and *in vivo* approaches to understand how gut-associated microbes impact host health.

## MATERIALS AND METHODS

### Generation of the individual bacterial metabolic models.

Genomes of Acetobacter fabarum (JOPD01000000), Acetobacter pomorum (JOKL01000000), Acetobacter tropicalis (JOKM01000000), Lactobacillus brevis (JOKA01000000), and Lactobacillus plantarum (JOJT01000000) were downloaded from NCBI and reannotated using the RAST annotation server (90). Two draft model reconstructions were generated for each genome and combined to generate a final model. The first models were obtained by performing reciprocal BLASTs of *Acetobacter* genomes against Escherichia coli strain K-12 substrain MG1655 and lactobacilli genomes against Lactobacillus plantarum strain WCFS1. Gene orthologs identified from the reciprocal blast searches were compared to the E. coli strain K-12 substrain MG1655 metabolic model iML1515 (91) and the Lactobacillus plantarum WCFS1 metabolic model (92). Then, reactions encoded by these genes were manually extracted to create a draft model. The second draft reconstructions were generated from the automated reconstruction pipeline ModelSEED (93) using the RAST reannotated genomes as input. For each bacterium, the two models were integrated and manually curated to remove redundant reactions and ensure correct reaction gene association, stoichiometry, and directionality. Organism-specific features and genes encoding metabolic reactions absent in Escherichia coli iML1515 and Lactobacillus plantarum WCFS1 metabolic models were identified by literature review and searches of BioCyc, KEGG, EcoCyc, BiGG, and BRENDA databases (94–98), and then added to the draft model. The models were further curated using nutrient utilization Biolog data (38) to verify and identify nutrient sources utilized by *Acetobacter* and *Lactobacillus*. All models were evaluated using MEMOTE (99).

### Model media composition.

All simulations were performed in one of three media types; a minimal medium, a base medium, and a rich medium. The minimal medium is a nutrient-poor medium containing glucose, glycerol, ammonia, sulfate, and phosphate as primary sources of carbon, nitrogen, sulfur, and phosphorus, respectively. Components of the minimal medium were selected to investigate the complete scope of interaction possible between *Drosophila* gut microbiota in the absence of host gut- and diet-derived nutrients. Components of the base medium were selected to match nutrient auxotrophies for all 5 bacteria and allow the growth of all 5 bacteria in isolation. Components of the rich medium comprised the complete set of nutrients required for growth by all five bacteria, all major sources of carbon, nitrogen, sulfur, and phosphorus for which these bacteria have annotated transporters, nutrient components of *Drosophila* holidic diet, and metabolites predicted to be available in the fly gut from a metabolomic analysis (100). All media nutrient constituents and the flux bounds used for all bacteria and all simulations are provided in Table S7 in the supplemental material.

### Model constraints applied.

Reaction fluxes for community members were obtained using the SteadyCom (40) flux variability analysis (FVA) implementation in the OpenCOBRA Toolbox (101) with the following constraints. Medoid growth rate vectors of individual species were computed by performing FVA while maintaining 99.99% of the maximum community growth rate (*μ*_max_) and simultaneously maximizing and minimizing flux through individual reactions to obtain individual species growth rate values for each simulation. For each species, we obtained between 1,000 and 5,000 growth rate values, representing species growth rates when each reaction was performing at its minimum and maximum while maintaining the maximum community growth rate. We used the medoid predicted growth rate value for each species as a lower bound for all subsequent model simulations by constraining SteadyCom parameters BMcon, BMrhs, and BMCsense to require each species to have a biomass value of at least the computed medoid in all simulations. A SteadyCom algorithm that minimizes the L1 (taxicab) norm of the predicted flux vectors was also applied to remove futile cycles and extraneous flux predictions.

### Model validation and comparison to empirical data.

Our models correctly reproduce usage patterns for 44 to 55% of the metabolites and compounds predicted to be utilized by *Drosophila* gut bacteria as characterized experimentally using Biolog plates (38). Comparisons between our model predictions of growth and Biolog data from Newell et al. (38) are provided at https://github.com/federatedcloud/DouglasMetabolicModels/tree/master/models/5.models_080719/Excel. Our predictions of metabolite release also match data from experimental studies for amino acids (30, 50) and B vitamins (39) from *Drosophila* gut microbiota. Last, published empirical data on *Drosophila* mono-associated with *Acetobacter* and *Lactobacillus*, e.g., Gould et al. (14) (Fig. 3B) and Newell et al. (38) (Fig. 1C) generally show three rankings in monococulture growth rates that are reproduced in our models for base and rich media: (i) monococulture *Lactobacillus* biomass and growth are higher than *Acetobacter* in the fly gut; (ii) comparable *Acetobacter* biomass and growth for different *Acetobacter* species; and (iii) relatively higher Lactobacillus brevis growth compared to Lactobacillus plantarum growth.

### Model simulation.

To find the maximum community growth rate we used the bisection algorithm in SteadyCom because it presented minimal convergence and feasibility issues with the constraints applied to our community model. Additionally, the default SteadyCom feasTol algorithm, which sets the allowed error for determining if an input solution is feasible, was set to 1e−8 for the solver. All parameters used to constrain SteadyCom simulations are available in the runSteadyCom and runSteadyComFVAMedoid functions.

### Analysis of simulated results.

Simulations were run for each subset of the 5 species, i.e., 2^5^−1 = 31 communities, 5 of which are single-species communities. We constructed the single-member communities as multispecies models to facilitate the analysis and simulation pipelines, particularly SteadyCom, which requires a multispecies model. We verified that growth rates for single-species models and the single-member community models with flux balance analysis (102) were identical (see https://github.com/federatedcloud/DouglasMetabolicModels/blob/v1.0.2/Tests/testMultiModelSingleton.m).

### Statistical analyses.

All statistical analyses were performed using R v.3.6.1 (103) with an alpha of 0.05 to assess significance. Bonferroni correction for multiple tests was applied where required. Statistical differences between the number of overlapping metabolites in different community sizes and metabolite use patterns associated with different ecological interactions were investigated by one-way analysis of variance (ANOVA) followed by Tukey’s honestly significant difference (HSD) *post hoc* test. Metabolite richness was calculated as the number of metabolites either consumed or released by a given taxon in each microbial treatment and medium combination. A mixed-effect two-way ANOVA was used to assess how the medium type (rich, basal, or minimal) and community size (number of taxa) influenced metabolite richness using the “lmer” function in lme4 package (104). The data for metabolite consumption and release were analyzed separately. Medium type and community size were included as categorical fixed effects and microbial treatment (combination of microbes in a community, e.g., AF + LB) and taxon (AF, AP, AT, LB, or LP) were designated categorical random effects. The “Anova” function in the car package (105) was used to perform a type III Wald’s F test to determine the effect of predictors with a Kenward-Rodger approximation to estimate residual degrees of freedom. A *post hoc* Tukey’s test was performed for each medium type to determine pairwise differences across community size. An analysis of deviance was performed to assess the significance of each random effect and the best linear unbiased prediction was estimated for each taxon using the “ranef” function to predict the global effect of each taxon across the different community size and medium combinations. Marginal and conditional *R*^2^ values were calculated using the MuMIn package (106).

A principal-component analysis (PCA) was performed to correlate metabolite consumption or release rates with community size, medium type, and microbial presence using the vegan package (107). A correlation matrix was implemented using the total sum rate of metabolites either consumed or released for each of the metabolite type bins (carbon, amino acids, nitrogen, etc.). The function “envfit” was used to correlate PC1 and PC2 with microbial presence with 999 permutations and significant vectors were plotted. In addition, a permutational multivariate analysis of variance (PERMANOVA) was used to assess the effect of community size by medium type as well as taxon on metabolite rates with the function “adonis.” Data were autoscaled and a Euclidean distance matrix was implemented for the model with 999 permutations. Consumption and release rates were analyzed separately for all analyses.

### Code and data availability.

All code used in this study can be found at https://github.com/federatedcloud/DouglasMetabolicModels/releases/tag/v1.0.2. The simulations were performed using v3.0.4 of the OpenCOBRA Toolbox and v7.5.1 of the Gurobi Optimizer (108). An optional, containerized environment for running the code is available at https://github.com/federatedcloud/COBRAContainers. All results derived from simulations can be found in DouglasMetabolicModels/analysis/CMP_and_CooperativeFluxes. A tutorial is provided for performing simulations in the repository’s top-level README file. The code is available under the MPL2 license.

SBML files of the models have been submitted to the BioModels database (109) with the following identifiers: MODEL2002040002, MODEL2002040003, MODEL2002040004, MODEL2002040005, and MODEL2002040006.

10.1128/mSystems.01369-20.7 TABLE S7 List of components in rich medium (A), base medium (B), or minimal medium (C). Download Table S7, PDF file, 0.2 MB. Copyright © 2021 Ankrah et al. 2021 Ankrah et al. https://creativecommons.org/licenses/by/4.0/ This content is distributed under the terms of the Creative Commons Attribution 4.0 International license.

10.1128/mSystems.01369-20.8 DATA SET 1 (A) Metabolite abbreviation key. (B) Predicted number of inputs and outputs from bacteria in rich medium. (C) Predicted number of inputs and outputs from bacteria in base medium. (D) Predicted number of inputs and outputs from bacteria in minimal medium. Download Data Set 1, XLSX file, 0.03 MB. Copyright © 2021 Ankrah et al. 2021 Ankrah et al. https://creativecommons.org/licenses/by/4.0/ This content is distributed under the terms of the Creative Commons Attribution 4.0 International license.

10.1128/mSystems.01369-20.9 DATA SET 2 Predicted metabolite flux available to the host. Download Data Set 2, XLSX file, 0.03 MB. Copyright © 2021 Ankrah et al. 2021 Ankrah et al. https://creativecommons.org/licenses/by/4.0/ This content is distributed under the terms of the Creative Commons Attribution 4.0 International license.
