# Supplementary material for: Predicted Metabolic Function of the Gut Microbiota of Drosophila melanogaster
Source: mSystems. 2021 May 4;6(3):e01369-20. doi: 10.1128/mSystems.01369-20 (PMC8269265; doi:10.1128/mSystems.01369-20)
Supplement: TABLE S1 [file msystems.01369-20-st001.pdf]

Table S1A. Predicted changes to bacterial growth (gdw h^{-1}) in co-culture compared to growth in monoculture - rich medium.

Community size	Community composition	Interaction type	Bacterium 1	Change in growth [a]	Bacterium 2	Change in growth [a]
2	AF-AP	Competitive	AF	-0.047543062	AP	-0.065061688
	AF-AT	Competitive	AF	-0.076238667	AT	-0.036366088
	AF-LB	Parasitic	AF	0.055074439	LB	-0.119156081
	AF-LP	Parasitic	AF	0.047204914	LP	-0.1135639
	AP-AT	Competitive	AP	-0.074874192	AT	-0.037730678
	AP-LB	Parasitic	AP	0.059915684	LB	-0.122596289
	AP-LP	Parasitic	AP	0.049206063	LP	-0.114985903
	AT-LB	Competitive	AT	-0.014359178	LB	-0.069815219
	AT-LP	Competitive	AT	-0.107234294	LP	-0.003816429
	LB-LP	Parasitic	LB	0.032214901	LP	-0.232262772
3	AF-AP-AT	Competitive	AF	-0.087082938	AP	-0.086340684
		Competitive	AF	-0.087082938	AT	-0.051786046
		Competitive	AP	-0.086340684	AT	-0.051786046
	AF-AP-LB	Competitive	AF	-0.002853789	AP	-0.048262407
		Competitive	AF	-0.002853789	LB	-0.123714149
		Competitive	AP	-0.048262407	LB	-0.123714149
	AF-AP-LP	Competitive	AF	-0.029562466	AP	-0.033782893
		Competitive	AF	-0.029562466	LP	-0.115023822
		Competitive	AP	-0.033782893	LP	-0.115023822
	AF-AT-LB	Parasitic	AF	-0.110219045	AT	0.152376904
		Competitive	AF	-0.110219045	LB	-0.189996451
		Parasitic	AT	0.152376904	LB	-0.189996451
	AF-AT-LP	Parasitic	AF	-0.107561003	AT	0.131553873
		Competitive	AF	-0.107561003	LP	-0.177088036
		Parasitic	AT	0.131553873	LP	-0.177088036
	AF-LB-LP	Parasitic	AF	0.06660369	LB	-0.221068991
		Parasitic	AF	0.06660369	LP	-0.106327846
		Competitive	LB	-0.221068991	LP	-0.106327846
	AP-AT-LB	Parasitic	AP	-0.106949154	AT	0.150741462
		Competitive	AP	-0.106949154	LB	-0.191157911
		Parasitic	AT	0.150741462	LB	-0.191157911
	AP-AT-LP	Parasitic	AP	-0.074220679	AT	0.102520301
		Competitive	AP	-0.074220679	LP	-0.180148566
		Parasitic	AT	0.102520301	LP	-0.180148566
	AP-LB-LP	Parasitic	AP	0.073164849	LB	-0.216806519
		Parasitic	AP	0.073164849	LP	-0.115252826
		Competitive	LB	-0.216806519	LP	-0.115252826
	AT-LB-LP	Competitive	AT	-0.028038695	LB	-0.156802785
		Competitive	AT	-0.028038695	LP	-0.103339428
		Competitive	LB	-0.156802785	LP	-0.103339428
4	AF-AP-AT-LB	Competitive	AF	-0.110695191	AP	-0.108231736
		Parasitic	AF	-0.110695191	AT	0.150034022
		Competitive	AF	-0.110695191	LB	-0.191100792
		Parasitic	AP	-0.108231736	AT	0.150034022
		Competitive	AP	-0.108231736	LB	-0.191100792
		Parasitic	AT	0.150034022	LB	-0.191100792
	AF-AP-AT-LP	Competitive	AF	-0.106694143	AP	-0.077213775
		Parasitic	AF	-0.106694143	AT	0.099597843
		Competitive	AF	-0.106694143	LP	-0.180144987
		Parasitic	AP	-0.077213775	AT	0.099597843
		Competitive	AP	-0.077213775	LP	-0.180144987
		Parasitic	AT	0.099597843	LP	-0.180144987
	AF-AP-LB-LP	Parasitic	AF	0.011016456	AP	-0.050282481
		Parasitic	AF	0.011016456	LB	-0.218304048
		Parasitic	AF	0.011016456	LP	-0.113878797

		Competitive	AP	-0.050282481	LB	-0.218304048
		Competitive	AP	-0.050282481	LP	-0.113878797
		Competitive	LB	-0.218304048	LP	-0.113878797
	AF-AT-LB-LP	Parasitic	AF	-0.109146218	AT	0.166705235
		Competitive	AF	-0.109146218	LB	-0.213566522
		Competitive	AF	-0.109146218	LP	-0.187422146
		Parasitic	AT	0.166705235	LB	-0.213566522
		Parasitic	AT	0.166705235	LP	-0.187422146
		Competitive	LB	-0.213566522	LP	-0.187422146
	AP-AT-LB-LP	Parasitic	AP	-0.106756685	AT	0.166029534
		Competitive	AP	-0.106756685	LB	-0.214101504
		Competitive	AP	-0.106756685	LP	-0.188105092
		Parasitic	AT	0.166029534	LB	-0.214101504
		Parasitic	AT	0.166029534	LP	-0.188105092
		Competitive	LB	-0.214101504	LP	-0.188105092
5	AF-AP-AT-LB-LP	Competitive	AF	-0.108186463	AP	-0.107792338
		Parasitic	AF	-0.108186463	AT	0.162643559
		Competitive	AF	-0.108186463	LB	-0.215194119
		Competitive	AF	-0.108186463	LP	-0.187010075
		Parasitic	AP	-0.107792338	AT	0.162643559
		Competitive	AP	-0.107792338	LB	-0.215194119
		Competitive	AP	-0.107792338	LP	-0.187010075
		Parasitic	AT	0.162643559	LB	-0.215194119
		Parasitic	AT	0.162643559	LP	-0.187010075
		Competitive	LB	-0.215194119	LP	-0.187010075

AF-*Acetobacter fabarum*; AP-*Acetobacter pomorum*; AT-*Acetobacter tropicalis*; LB-*Lactobacillus brevis*; LP-*Lactobacillus plantarum*

[a]Change in growth is calculated by subtracting growth of a microbe in mono-culture to growth of the microbe in co-culture.

Table S1B. Predicted changes to bacterial growth (gdw h^{-1}) in co-culture compared to growth in isolation - base medium.

Community size	Community composition	Interaction type	Bacterium 1	Change in growth [a]	Bacterium 2	Change in growth [a]
2	AF-AP	Competitive	AF	-0.001884309	AP	-0.009319108
	AF-AT	Competitive	AF	-0.009198881	AT	-0.002004535
	AF-LB	Competitive	AF	-0.007072457	LB	-0.002948602
	AF-LP	Parasitic	AF	0.000173024	LP	-0.008120277
	AP-AT	Competitive	AP	-0.008869707	AT	-0.002333709
	AP-LB	Competitive	AP	-0.007072456	LB	-0.002948602
	AP-LP	Parasitic	AP	0.000247659	LP	-0.008173545
	AT-LB	Competitive	AT	-0.009023334	LB	-0.001556102
	AT-LP	Parasitic	AT	0.001117801	LP	-0.008794637
	LB-LP	Competitive	LB	-0.001573434	LP	-0.014420165
3	AF-AP-AT	Competitive	AF	-0.009702916	AP	-0.009721573
		Competitive	AF	-0.009702916	AT	-0.002982338
		Competitive	AP	-0.009721573	AT	-0.002982338
	AF-AP-LB	Competitive	AF	-0.004808531	AP	-0.010774966
		Competitive	AF	-0.004808531	LB	-0.004870388
		Competitive	AP	-0.010774966	LB	-0.004870388
	AF-AP-LP	Competitive	AF	-0.005672564	AP	-0.005215904
		Competitive	AF	-0.005672564	LP	-0.008221575
		Competitive	AP	-0.005215904	LP	-0.008221575
	AF-AT-LB	Parasitic	AF	-0.010831436	AT	0.014005427
		Competitive	AF	-0.010831436	LB	-0.018259108
		Parasitic	AT	0.014005427	LB	-0.018259108
	AF-AT-LP	Parasitic	AF	-0.010836787	AT	0.006768986
		Competitive	AF	-0.010836787	LP	-0.013090051
		Parasitic	AT	0.006768986	LP	-0.013090051
	AF-LB-LP	Parasitic	AF	0.00453964	LB	-0.016130989

		Parasitic	AF	0.00453964	LP	-0.011099678
		Competitive	LB	-0.016130989	LP	-0.011099678
	AP-AT-LB	Parasitic	AP	-0.010833167	AT	0.014395807
		Competitive	AP	-0.010833167	LB	-0.018536512
		Parasitic	AT	0.014395807	LB	-0.018536512
	AP-AT-LP	Parasitic	AP	-0.010835518	AT	0.007082089
		Competitive	AP	-0.010835518	LP	-0.013314428
		Parasitic	AT	0.007082089	LP	-0.013314428
	AP-LB-LP	Parasitic	AP	0.004762498	LB	-0.015717421
		Parasitic	AP	0.004762498	LP	-0.01167233
		Competitive	LB	-0.015717421	LP	-0.01167233
	AT-LB-LP	Competitive	AT	-0.005040301	LB	-0.017196784
		Competitive	AT	-0.005040301	LP	-0.003195905
		Competitive	LB	-0.017196784	LP	-0.003195905
4	AF-AP-AT-LB	Competitive	AF	-0.010834665	AP	-0.010850631
		Parasitic	AF	-0.010834665	AT	0.013922938
		Competitive	AF	-0.010834665	LB	-0.018449745
		Parasitic	AP	-0.010850631	AT	0.013922938
		Competitive	AP	-0.010850631	LB	-0.018449745
		Parasitic	AT	0.013922938	LB	-0.018449745
	AF-AP-AT-LP	Competitive	AF	-0.010850423	AP	-0.010839312
		Parasitic	AF	-0.010850423	AT	0.006582039
		Competitive	AF	-0.010850423	LP	-0.013206757
		Parasitic	AP	-0.010839312	AT	0.006582039
		Competitive	AP	-0.010839312	LP	-0.013206757
		Parasitic	AT	0.006582039	LP	-0.013206757
	AF-AP-LB-LP	Competitive	AF	-0.003309859	AP	-0.003135658
		Competitive	AF	-0.003309859	LB	-0.01575855
		Competitive	AF	-0.003309859	LP	-0.01162791
		Competitive	AP	-0.003135658	LB	-0.01575855
		Competitive	AP	-0.003135658	LP	-0.01162791
		Competitive	LB	-0.01575855	LP	-0.01162791
	AF-AT-LB-LP	Parasitic	AF	-0.010848141	AT	0.013942745
		Competitive	AF	-0.010848141	LB	-0.018583218
		Competitive	AF	-0.010848141	LP	-0.01561282
		Parasitic	AT	0.013942745	LB	-0.018583218
		Parasitic	AT	0.013942745	LP	-0.01561282
		Competitive	LB	-0.018583218	LP	-0.01561282
	AP-AT-LB-LP	Parasitic	AP	-0.010838311	AT	0.014321286
		Competitive	AP	-0.010838311	LB	-0.0188563
		Competitive	AP	-0.010838311	LP	-0.01561693
		Parasitic	AT	0.014321286	LB	-0.0188563
		Parasitic	AT	0.014321286	LP	-0.01561693
		Competitive	LB	-0.0188563	LP	-0.01561693
5	AF-AP-AT-LB-LP	Competitive	AF	-0.010850631	AP	-0.01083872
		Parasitic	AF	-0.010850631	AT	0.013847162
		Competitive	AF	-0.010850631	LB	-0.018770392
		Competitive	AF	-0.010850631	LP	-0.015615938
		Parasitic	AP	-0.01083872	AT	0.013847162
		Competitive	AP	-0.01083872	LB	-0.018770392
		Competitive	AP	-0.01083872	LP	-0.015615938
		Parasitic	AT	0.013847162	LB	-0.018770392
		Parasitic	AT	0.013847162	LP	-0.015615938
		Competitive	LB	-0.018770392	LP	-0.015615938

AF-*Acetobacter fabarum*; AP-*Acetobacter pomorum*; AT-*Acetobacter tropicalis*; LB-*Lactobacillus brevis*; LP-*Lactobacillus plantarum*

[a] Change in growth is calculated by subtracting growth of a microbe in mono-culture to growth of the microbe in co-culture.

Table S1C. Predicted changes to bacterial growth (gdw h^{-1}) in co-culture compared to growth in isolation - minimal medium.

Community size	Community composition	Interaction type	Bacterium 1	Change in growth [a]	Bacterium 2	Change in growth [a]
2	AF-AP	Parasitic	AF	0.004306849	AP	-0.004306849
	AF-AT	Parasitic	AF	0.000426858	AT	-0.000426858
	AF-LB	Neutral	AF	0	LB	0
	AF-LP	Mutualistic	AF	0.003271789	LP	0.002523747
	AP-AT	Competitive	AP	-0.004216446	AT	-0.000438274
	AP-LB	Amensal	AP	-0.00465472	LB	0
	AP-LP	Parasitic	AP	-0.001375683	LP	0.00251052
	AT-LB	Amensal	AT	-0.00465472	LB	0
	AT-LP	Parasitic	AT	-0.001614864	LP	0.002947009
	LB-LP	Neutral	LB	0	LP	0
3	AF-AP-AT	Parasitic	AF	0.000404493	AP	-0.004250006
		Parasitic	AF	0.000404493	AT	-0.000809207
		Competitive	AP	-0.004250006	AT	-0.000809207
	AF-AP-LB	Amensal	AF	0	AP	-0.00465472
		Neutral	AF	0	LB	0
		Amensal	AP	-0.00465472	LB	0
	AF-AP-LP	Parasitic	AF	0.002786746	AP	-0.004291046
		Mutual	AF	0.002786746	LP	0.002745237
		Parasitic	AP	-0.004291046	LP	0.002745237
	AF-AT-LB	Amensal	AF	0	AT	-0.00465472
		Neutral	AF	0	LB	0
		Amensal	AT	-0.00465472	LB	0
	AF-AT-LP	Parasitic	AF	0.000355333	AT	-0.00429841
		Mutual	AF	0.000355333	LP	0.007195837
		Parasitic	AT	-0.00429841	LP	0.007195837
	AF-LB-LP	Mutual	AF	0.003216672	LB	0.000451943
		Mutual	AF	0.003216672	LP	0.002168724
		Mutual	LB	0.000451943	LP	0.002168724
	AP-AT-LB	Competitive	AP	-0.00465472	AT	-0.00465472
		Amensal	AP	-0.00465472	LB	0
		Amensal	AT	-0.00465472	LB	0
	AP-AT-LP	Competitive	AP	-0.004301871	AT	-0.004301009
		Parasitic	AP	-0.004301871	LP	0.007205113
		Parasitic	AT	-0.004301009	LP	0.007205113
	AP-LB-LP	Parasitic	AP	-0.001419561	LB	0.000449321
		Parasitic	AP	-0.001419561	LP	0.002137628
		Mutual	LB	0.000449321	LP	0.002137628
	AT-LB-LP	Parasitic	AT	-0.003674414	LB	0.003541452
		Parasitic	AT	-0.003674414	LP	0.003135363
		Mutual	LB	0.003541452	LP	0.003135363
4	AF-AP-AT-LB	Amensal	AF	0	AP	-0.00465472
		Amensal	AF	0	AT	-0.00465472
		Neutral	AF	0	LB	0
		Competitive	AP	-0.00465472	AT	-0.00465472
		Amensal	AP	-0.00465472	LB	0
		Amensal	AT	-0.00465472	LB	0
	AF-AP-AT-LP	Parasitic	AF	0.000352853	AP	-0.004301867
		Parasitic	AF	0.000352853	AT	-0.004301188
		Mutual	AF	0.000352853	LP	0.006561499
		Competitive	AP	-0.004301867	AT	-0.004301188
		Parasitic	AP	-0.004301867	LP	0.006561499
		Parasitic	AT	-0.004301188	LP	0.006561499
	AF-AP-LB-LP	Parasitic	AF	0.002791665	AP	-0.004269056
		Mutual	AF	0.002791665	LB	0.000377067

		Mutual	AF	0.002791665	LP	0.002316005
		Parasitic	AP	-0.004269056	LB	0.000377067
		Parasitic	AP	-0.004269056	LP	0.002316005
		Mutual	LB	0.000377067	LP	0.002316005
	AF-AT-LB-LP	Parasitic	AF	0.000355324	AT	-0.004299397
		Mutual	AF	0.000355324	LB	0.000481636
		Mutual	AF	0.000355324	LP	0.006712112
		Parasitic	AT	-0.004299397	LB	0.000481636
		Parasitic	AT	-0.004299397	LP	0.006712112
		Mutual	LB	0.000481636	LP	0.006712112
	AP-AT-LB-LP	Competitive	AP	-0.004301471	AT	-0.004301906
		Parasitic	AP	-0.004301471	LB	0.000478799
		Parasitic	AP	-0.004301471	LP	0.006723336
		Parasitic	AT	-0.004301906	LB	0.000478799
		Parasitic	AT	-0.004301906	LP	0.006723336
		Mutual	LB	0.000478799	LP	0.006723336
5	AF-AP-AT-LB-LP	Parasitic	AF	0.000352834	AP	-0.004301887
		Parasitic	AF	0.000352834	AT	-0.004301887
		Mutual	AF	0.000352834	LB	0.000474397
		Mutual	AF	0.000352834	LP	0.006084601
		Competitive	AP	-0.004301887	AT	-0.004301887
		Parasitic	AP	-0.004301887	LB	0.000474397
		Parasitic	AP	-0.004301887	LP	0.006084601
		Parasitic	AT	-0.004301887	LB	0.000474397
		Parasitic	AT	-0.004301887	LP	0.006084601
		Mutual	LB	0.000474397	LP	0.006084601

AF-*Acetobacter fabarum*; AP-*Acetobacter pomorum*; AT-*Acetobacter tropicalis*; LB-*Lactobacillus brevis*; LP-*Lactobacillus plantarum*

[a] Change in growth is calculated by subtracting growth of a microbe in mono-culture to growth of the microbe in co-culture.
