# Supplementary material for: Predicted Metabolic Function of the Gut Microbiota of Drosophila melanogaster
Source: mSystems. 2021 May 4;6(3):e01369-20. doi: 10.1128/mSystems.01369-20 (PMC8269265; doi:10.1128/mSystems.01369-20)
Supplement: TABLE S2 [file msystems.01369-20-st002.pdf]

Table S2A. Predicted number of inputs and outputs from bacteria - rich medium.

Community	Number of species	Organism	Number of overlapping inputs	Number of overlapping outputs	Total input count	Total output count
AF	1	AF	0	0	29	6
AF_AP	2	AF	24	6	28	6
AF_AP	2	AP	24	6	26	6
AF_AP_AT	3	AF	24	6	26	6
AF_AP_AT	3	AP	24	6	26	6
AF_AP_AT	3	AT	19	6	26	9
AF_AP_AT_LB	4	AF	21	5	24	6
AF_AP_AT_LB	4	AP	24	5	26	6
AF_AP_AT_LB	4	AT	24	4	36	7
AF_AP_AT_LB	4	LB	12	1	40	12
AF_AP_AT_LB_LP	5	AF	23	5	25	5
AF_AP_AT_LB_LP	5	AP	25	5	26	6
AF_AP_AT_LB_LP	5	AT	25	4	37	6
AF_AP_AT_LB_LP	5	LB	25	3	37	8
AF_AP_AT_LB_LP	5	LP	27	2	39	5
AF_AP_AT_LP	4	AF	24	5	25	5
AF_AP_AT_LP	4	AP	25	5	26	6
AF_AP_AT_LP	4	AT	22	4	32	8
AF_AP_AT_LP	4	LP	11	1	36	7
AF_AP_LB	3	AF	25	5	28	5
AF_AP_LB	3	AP	24	5	27	6
AF_AP_LB	3	LB	10	1	41	12
AF_AP_LB_LP	4	AF	25	5	28	5
AF_AP_LB_LP	4	AP	23	5	26	6
AF_AP_LB_LP	4	LB	25	4	36	10
AF_AP_LB_LP	4	LP	29	2	46	5
AF_AP_LP	3	AF	26	6	27	6
AF_AP_LP	3	AP	26	6	30	7
AF_AP_LP	3	LP	13	0	44	7
AF_AT	2	AF	19	6	26	6
AF_AT	2	AT	19	6	25	9
AF_AT_LB	3	AF	19	6	24	6
AF_AT_LB	3	AT	20	6	35	8
AF_AT_LB	3	LB	12	1	40	12
AF_AT_LB_LP	4	AF	21	4	25	5
AF_AT_LB_LP	4	AT	23	4	37	6
AF_AT_LB_LP	4	LB	25	3	37	8
AF_AT_LB_LP	4	LP	27	2	39	5
AF_AT_LP	3	AF	22	5	25	6
AF_AT_LP	3	AT	21	5	33	9
AF_AT_LP	3	LP	11	1	36	7
AF_LB	2	AF	10	1	30	6
AF_LB	2	LB	10	1	41	12
AF_LB_LP	3	AF	15	2	29	5
AF_LB_LP	3	LB	25	4	36	10
AF_LB_LP	3	LP	29	2	46	5
AF_LP	2	AF	11	0	30	7
AF_LP	2	LP	11	0	44	7
AP	1	AP	0	0	29	6
AP_AT	2	AP	19	6	27	6
AP_AT	2	AT	19	6	26	9
AP_AT_LB	3	AP	23	4	26	6
AP_AT_LB	3	AT	25	4	36	7
AP_AT_LB	3	LB	11	1	40	12

AP_AT_LB_LP	4	AP	22	4	25	6
AP_AT_LB_LP	4	AT	25	4	37	6
AP_AT_LB_LP	4	LB	26	3	37	8
AP_AT_LB_LP	4	LP	28	2	40	5
AP_AT_LP	3	AP	22	4	26	6
AP_AT_LP	3	AT	23	4	33	8
AP_AT_LP	3	LP	11	1	36	7
AP_LB	2	AP	9	1	30	6
AP_LB	2	LB	9	1	41	12
AP_LB_LP	3	AP	12	2	28	6
AP_LB_LP	3	LB	25	4	36	10
AP_LB_LP	3	LP	28	2	45	5
AP_LP	2	AP	12	0	30	7
AP_LP	2	LP	12	0	45	7
AT	1	AT	0	0	26	8
AT_LB	2	AT	9	1	26	7
AT_LB	2	LB	9	1	41	10
AT_LB_LP	3	AT	12	1	29	7
AT_LB_LP	3	LB	27	3	38	8
AT_LB_LP	3	LP	28	2	42	4
AT_LP	2	AT	13	1	28	7
AT_LP	2	LP	13	1	44	6
LB	1	LB	0	0	45	9
LB_LP	2	LB	15	4	45	9
LB_LP	2	LP	15	4	30	6
LP	1	LP	0	0	44	6

AF-*Acetobacter fabarum*; AP-*Acetobacter pomorum*; AT-*Acetobacter tropicalis*; LB-*Lactobacillus brevis*; LP-*Lactobacillus plantarum*

Table S2B. Predicted number of inputs and outputs from bacteria - base medium.

Community	Number of species	Organism	Number of overlapping inputs	Number of overlapping outputs	Total input count	Total output count
AF	1	AF	0	0	15	6
AF_AP	2	AF	13	6	14	6
AF_AP	2	AP	13	6	14	6
AF_AP_AT	3	AF	15	4	20	5
AF_AP_AT	3	AP	15	4	21	5
AF_AP_AT	3	AT	12	4	15	12
AF_AP_AT_LB	4	AF	17	4	22	5
AF_AP_AT_LB	4	AP	18	4	22	5
AF_AP_AT_LB	4	AT	18	4	22	9
AF_AP_AT_LB	4	LB	10	1	25	13
AF_AP_AT_LB_LP	5	AF	17	4	22	5
AF_AP_AT_LB_LP	5	AP	18	4	21	6
AF_AP_AT_LB_LP	5	AT	18	5	22	9
AF_AP_AT_LB_LP	5	LB	19	5	25	13
AF_AP_AT_LB_LP	5	LP	21	6	25	7
AF_AP_AT_LP	4	AF	16	4	19	5
AF_AP_AT_LP	4	AP	16	4	19	5
AF_AP_AT_LP	4	AT	15	5	18	9
AF_AP_AT_LP	4	LP	12	1	24	7
AF_AP_LB	3	AF	19	6	21	7
AF_AP_LB	3	AP	18	5	21	6
AF_AP_LB	3	LB	11	2	26	11
AF_AP_LB_LP	4	AF	20	7	22	7
AF_AP_LB_LP	4	AP	20	7	21	7
AF_AP_LB_LP	4	LB	17	2	25	13

AF_AP_LB_LP	4	LP	18	1	28	5
AF_AP_LP	3	AF	18	7	19	7
AF_AP_LP	3	AP	19	7	20	8
AF_AP_LP	3	LP	12	0	25	6
AF_AT	2	AF	12	4	19	5
AF_AT	2	AT	12	4	15	11
AF_AT_LB	3	AF	16	4	22	5
AF_AT_LB	3	AT	17	4	22	9
AF_AT_LB	3	LB	10	1	25	13
AF_AT_LB_LP	4	AF	16	4	22	5
AF_AT_LB_LP	4	AT	18	5	22	9
AF_AT_LB_LP	4	LB	19	5	25	12
AF_AT_LB_LP	4	LP	21	6	26	8
AF_AT_LP	3	AF	16	4	20	5
AF_AT_LP	3	AT	15	5	18	9
AF_AT_LP	3	LP	12	1	24	7
AF_LB	2	AF	12	2	21	7
AF_LB	2	LB	12	2	27	10
AF_LB_LP	3	AF	12	3	23	7
AF_LB_LP	3	LB	17	2	25	13
AF_LB_LP	3	LP	18	1	28	5
AF_LP	2	AF	12	0	20	8
AF_LP	2	LP	12	0	25	6
AP	1	AP	0	0	14	7
AP_AT	2	AP	12	5	21	5
AP_AT	2	AT	12	5	15	12
AP_AT_LB	3	AP	16	4	21	5
AP_AT_LB	3	AT	17	4	22	8
AP_AT_LB	3	LB	10	1	25	13
AP_AT_LB_LP	4	AP	16	4	20	5
AP_AT_LB_LP	4	AT	18	5	22	7
AP_AT_LB_LP	4	LB	19	5	25	13
AP_AT_LB_LP	4	LP	21	6	26	7
AP_AT_LP	3	AP	16	4	20	5
AP_AT_LP	3	AT	15	5	18	9
AP_AT_LP	3	LP	12	1	24	7
AP_LB	2	AP	12	2	21	7
AP_LB	2	LB	12	2	27	10
AP_LB_LP	3	AP	12	2	22	7
AP_LB_LP	3	LB	17	1	25	12
AP_LB_LP	3	LP	18	1	28	5
AP_LP	2	AP	12	0	20	8
AP_LP	2	LP	12	0	25	6
AT	1	AT	0	0	16	6
AT_LB	2	AT	11	1	20	6
AT_LB	2	LB	11	1	27	9
AT_LB_LP	3	AT	14	1	20	6
AT_LB_LP	3	LB	17	1	25	12
AT_LB_LP	3	LP	20	0	31	3
AT_LP	2	AT	12	0	19	7
AT_LP	2	LP	12	0	24	5
LB	1	LB	0	0	27	4
LB_LP	2	LB	18	0	27	10
LB_LP	2	LP	18	0	30	2
LP	1	LP	0	0	22	4

AF-*Acetobacter fabarum*; AP-*Acetobacter pomorum*; AT-*Acetobacter tropicalis*; LB-*Lactobacillus brevis*; LP-*Lactobacillus plantarum*

**Table S2C. Predicted number of inputs and outputs from bacteria - minimal medium.**						
**Community**	**Number of species**	**Organism**	**Number of overlapping inputs**	**Number of overlapping outputs**	**Total input count**	**Total output count**
AF	1	AF	0	0	0	0
AF_AP	2	AF	4	1	9	7
AF_AP	2	AP	4	1	11	5
AF_AP_AT	3	AF	8	1	18	1
AF_AP_AT	3	AP	8	1	16	2
AF_AP_AT	3	AT	3	1	5	17
AF_AP_AT_LB	4	AF	0	0	0	0
AF_AP_AT_LB	4	AP	0	0	0	0
AF_AP_AT_LB	4	AT	0	0	0	0
AF_AP_AT_LB	4	LB	0	0	0	0
AF_AP_AT_LB_LP	5	AF	12	5	14	10
AF_AP_AT_LB_LP	5	AP	12	5	12	13
AF_AP_AT_LB_LP	5	AT	11	4	12	10
AF_AP_AT_LB_LP	5	LB	11	1	28	6
AF_AP_AT_LB_LP	5	LP	8	1	18	18
AF_AP_AT_LP	4	AF	11	4	14	7
AF_AP_AT_LP	4	AP	11	4	13	8
AF_AP_AT_LP	4	AT	10	5	11	11
AF_AP_AT_LP	4	LP	4	1	16	10
AF_AP_LB	3	AF	0	0	0	0
AF_AP_LB	3	AP	0	0	0	0
AF_AP_LB	3	LB	0	0	0	0
AF_AP_LB_LP	4	AF	11	2	12	10
AF_AP_LB_LP	4	AP	11	3	12	11
AF_AP_LB_LP	4	LB	9	1	27	5
AF_AP_LB_LP	4	LP	9	1	15	18
AF_AP_LP	3	AF	10	2	15	8
AF_AP_LP	3	AP	11	3	12	10
AF_AP_LP	3	LP	4	1	14	10
AF_AT	2	AF	3	1	17	1
AF_AT	2	AT	3	1	4	14
AF_AT_LB	3	AF	0	0	0	0
AF_AT_LB	3	AT	0	0	0	0
AF_AT_LB	3	LB	0	0	0	0
AF_AT_LB_LP	4	AF	11	7	15	9
AF_AT_LB_LP	4	AT	10	8	11	20
AF_AT_LB_LP	4	LB	12	1	27	7
AF_AT_LB_LP	4	LP	8	1	19	17
AF_AT_LP	3	AF	10	6	14	9
AF_AT_LP	3	AT	10	6	12	17
AF_AT_LP	3	LP	3	0	17	9
AF_LB	2	AF	0	0	0	0
AF_LB	2	LB	0	0	0	0
AF_LB_LP	3	AF	9	3	14	19
AF_LB_LP	3	LB	10	1	27	4
AF_LB_LP	3	LP	9	2	15	19
AF_LP	2	AF	3	0	13	13
AF_LP	2	LP	3	0	14	10
AP	1	AP	0	0	5	2
AP_AT	2	AP	3	1	16	2
AP_AT	2	AT	3	1	5	13
AP_AT_LB	3	AP	0	0	0	0
AP_AT_LB	3	AT	0	0	0	0
AP_AT_LB	3	LB	0	0	0	0

AP_AT_LB_LP	4	AP	12	4	12	11
AP_AT_LB_LP	4	AT	11	4	12	13
AP_AT_LB_LP	4	LB	10	1	28	6
AP_AT_LB_LP	4	LP	8	1	17	18
AP_AT_LP	3	AP	11	6	13	9
AP_AT_LP	3	AT	10	5	12	14
AP_AT_LP	3	LP	4	1	15	10
AP_LB	2	AP	0	0	0	0
AP_LB	2	LB	0	0	0	0
AP_LB_LP	3	AP	8	2	11	18
AP_LB_LP	3	LB	9	2	28	5
AP_LB_LP	3	LP	9	2	15	17
AP_LP	2	AP	3	0	10	13
AP_LP	2	LP	3	0	14	7
AT	1	AT	0	0	4	2
AT_LB	2	AT	0	0	0	0
AT_LB	2	LB	0	0	0	0
AT_LB_LP	3	AT	11	2	13	18
AT_LB_LP	3	LB	12	0	28	7
AT_LB_LP	3	LP	9	2	18	16
AT_LP	2	AT	3	0	12	14
AT_LP	2	LP	3	0	15	9
LB	1	LB	0	0	0	0
LB_LP	2	LB	0	0	0	0
LB_LP	2	LP	0	0	0	0
LP	1	LP	0	0	0	0

AF-*Acetobacter fabarum*; AP-*Acetobacter pomorum*; AT-*Acetobacter tropicalis*; LB-*Lactobacillus brevis*; LP-*Lactobacillus plantarum*

Table S2D. Summary statistics for Figure 2B

	f.value	p.value	Tukey comparison	p.value
**Rich**	27.11	6.27E-14	four-five	0.9457374
			one-five	0
			three-five	0.3474896
			two-five	0.0005505
			one-four	0
			three-four	0.3658145
			two-four	0.0000039
			three-one	0
			two-one	0.0000047
			two-three	0.0005415
**Base**	56.06	2.00E-16	four-five	0.8101439
			one-five	0
			three-five	0.0438476
			two-five	0.0000622
			one-four	0
			three-four	0.0410586
			two-four	0.0000008
			three-one	0
			two-one	0
			two-three	0.0043816
**Minimal**	12.19	0.00000011	four-five	0.5243205
			one-five	0.0001562
			three-five	0.0606031
			two-five	0.0000753
			one-four	0.0005432
			three-four	0.359816

	two-four	0.0000223
	three-one	0.0120029
	two-one	0.8429174
	two-three	0.002909
