# Supplementary material for: Predicted Metabolic Function of the Gut Microbiota of Drosophila melanogaster
Source: mSystems. 2021 May 4;6(3):e01369-20. doi: 10.1128/mSystems.01369-20 (PMC8269265; doi:10.1128/mSystems.01369-20)
Supplement: TABLE S3 [file msystems.01369-20-st003.pdf]

Table S3A. Predicted community metabolite use patterns for competitive, parasitic and mutualistic interactions - rich medium.

Community size	Community composition	Pairwise comparison	Interaction Type	Single-use	Co-consumed	Cross-fed	Single-produced	Co-produced
2	AF_AP	AF-AP	Competitive	4	25	0	0	6
	AF_AT	AF-AT	Competitive	10	20	1	2	6
	AF_LB	AF-LB	Parasitic	43	11	6	10	1
	AF_LP	AF-LP	Parasitic	43	12	7	7	0
	AP_AT	AP-AT	Competitive	11	20	2	1	6
	AP_LB	AP-LB	Parasitic	44	10	7	9	1
	AP_LP	AP-LP	Parasitic	42	13	7	7	0
	AT_LB	AT-LB	Competitive	44	10	3	12	1
	AT_LP	AT-LP	Competitive	41	14	3	8	1
	LB_LP	LB-LP	Parasitic	35	18	4	3	4
3	AF-AP-AT	AF-AP	Competitive	2	25	0	0	6
		AF-AT	Competitive	11	20	1	2	6
		AP-AT	Competitive	11	20	1	2	6
	AF-AP-LB	AF-AP	Competitive	5	25	0	1	5
		AF-LB	Competitive	41	11	6	9	1
		AP-LB	Competitive	42	10	6	10	1
	AF-AP-LP	AF-AP	Competitive	5	26	0	1	6
		AF-LP	Competitive	38	13	7	6	0
		AP-LP	Competitive	41	13	7	7	0
	AF-AT-LB	AF-AT	Parasitic	18	20	1	1	6
		AF-LB	Competitive	37	11	5	11	1
		AT-LB	Parasitic	46	12	5	13	1
	AF-AT-LP	AF-AT	Parasitic	13	22	1	4	5
		AF-LP	Competitive	33	12	4	7	1
		AT-LP	Parasitic	40	11	7	7	1
	AF-LB-LP	AF-LB	Parasitic	36	12	5	6	2
		AF-LP	Parasitic	39	16	4	6	0
		LB-LP	Competitive	21	28	5	6	2
	AP-AT-LB	AP-AT	Parasitic	13	24	1	4	4
		AP-LB	Competitive	39	10	7	9	1
		AT-LB	Parasitic	46	12	6	11	1
	AP-AT-LP	AP-AT	Parasitic	12	23	1	5	4
		AP-LP	Competitive	36	11	4	7	1
		AT-LP	Parasitic	38	12	7	6	1
	AP-LB-LP	AP-LB	Parasitic	39	10	5	7	2
		AP-LP	Parasitic	43	13	4	7	0
		LB-LP	Competitive	20	28	5	6	2
	AT-LB-LP	AT-LB	Competitive	41	11	4	9	1
		AT-LP	Competitive	43	12	4	7	0
		LB-LP	Competitive	22	27	4	4	2
4	AF-AP-AT-LB	AF-AP	Competitive	4	23	0	2	5
		AF-AT	Parasitic	17	21	1	2	5
		AF-LB	Competitive	37	11	5	11	1
		AP-AT	Parasitic	13	24	1	4	4
		AP-LB	Competitive	39	10	7	9	1
		AT-LB	Parasitic	46	12	6	11	1
	AF-AP-AT-LP	AF-AP	Competitive	3	24	0	1	5
		AF-AT	Parasitic	14	21	1	4	4
		AF-LP	Competitive	33	12	4	6	1
		AP-AT	Parasitic	11	23	1	5	4
		AP-LP	Competitive	36	11	4	7	1
		AT-LP	Parasitic	39	11	7	6	1
	AF-AP-LB-LP	AF-AP	Parasitic	6	24	0	1	5
		AF-LB	Parasitic	35	12	5	6	2

		AF-LP	Parasitic	38	16	4	6	0
		AP-LB	Competitive	37	10	5	7	2
		AP-LP	Competitive	40	14	4	7	0
		LB-LP	Competitive	21	28	5	6	2
	AF-AT-LB-LP	AF-AT	Parasitic	18	22	0	3	4
		AF-LB	Competitive	36	11	4	7	1
		AF-LP	Competitive	34	13	4	6	0
		AT-LB	Parasitic	47	12	3	9	1
		AT-LP	Parasitic	44	14	4	7	0
		LB-LP	Competitive	18	27	4	5	2
	AP-AT-LB-LP	AP-AT	Parasitic	16	23	0	4	4
		AP-LB	Competitive	37	10	5	7	1
		AP-LP	Competitive	37	12	4	7	0
		AT-LB	Parasitic	47	12	3	9	1
		AT-LP	Parasitic	45	14	4	7	0
		LB-LP	Competitive	17	28	4	5	2
5	AF-AP-AT-LB-LP	AF-AP	Competitive	3	24	0	1	5
		AF-AT	Parasitic	18	22	0	3	4
		AF-LB	Competitive	36	11	4	7	1
		AF-LP	Competitive	35	13	3	7	0
		AP-AT	Parasitic	15	24	0	4	4
		AP-LB	Competitive	36	11	5	7	1
		AP-LP	Competitive	36	13	3	8	0
		AT-LB	Parasitic	47	12	3	9	1
		AT-LP	Parasitic	44	14	4	7	0
		LB-LP	Competitive	18	27	4	5	2

AF-*Acetobacter fabarum*; AP-*Acetobacter pomorum*; AT-*Acetobacter tropicalis*; LB-*Lactobacillus brevis*; LP-*Lactobacillus plantarum*

Table S3B. Predicted community metabolite use patterns for competitive, parasitic and mutualistic interactions - base medium

Community size	Community composition	Pairwise comparison	Interaction Type	Single-use	Co-consumed	Cross-fed	Single-produced	Co-produced
2	AF_AP	AF-AP	Competitive	0	14	0	0	6
	AF_AT	AF-AT	Competitive	3	13	5	1	5
	AF_LB	AF-LB	Competitive	17	13	5	8	2
	AF_LP	AF-LP	Parasitic	12	13	7	5	1
	AP_AT	AP-AT	Competitive	4	13	6	1	5
	AP_LB	AP-LB	Competitive	17	13	5	8	2
	AP_LP	AP-LP	Parasitic	12	13	7	5	1
	AT_LB	AT-LB	Competitive	18	12	5	8	1
	AT_LP	AT-LP	Parasitic	12	13	5	5	1
	LB_LP	LB-LP	Competitive	7	21	8	4	0
3	AF-AP-AT	AF-AP	Competitive	1	20	0	0	5
		AF-AT	Competitive	4	13	5	2	5
		AP-AT	Competitive	4	13	6	1	5
	AF-AP-LB	AF-AP	Competitive	0	21	0	1	6
		AF-LB	Competitive	17	12	6	8	2
		AP-LB	Competitive	17	12	6	9	1
	AF-AP-LP	AF-AP	Competitive	1	19	0	1	7
		AF-LP	Competitive	14	12	6	5	1
		AP-LP	Competitive	12	13	7	5	1
	AF-AT-LB	AF-AT	Parasitic	5	18	3	1	5
		AF-LB	Competitive	21	10	6	10	1
		AT-LB	Parasitic	17	11	8	12	1
	AF-AT-LP	AF-AT	Parasitic	2	18	0	4	5
		AF-LP	Competitive	14	13	4	6	1
		AT-LP	Parasitic	13	12	5	7	2

	AF-LB-LP	AF-LB	Parasitic	22	11	4	12	2
		AF-LP	Parasitic	23	12	4	6	1
		LB-LP	Competitive	6	19	9	9	0
	AP-AT-LB	AP-AT	Parasitic	5	18	2	1	5
		AP-LB	Competitive	20	10	6	10	1
		AT-LB	Parasitic	17	11	8	11	1
	AP-AT-LP	AP-AT	Parasitic	2	18	0	4	5
		AP-LP	Competitive	14	13	4	6	1
		AT-LP	Parasitic	13	12	5	7	2
	AP-LB-LP	AP-LB	Parasitic	21	11	4	13	1
		AP-LP	Parasitic	22	12	4	6	1
		LB-LP	Competitive	6	19	9	8	0
	AT-LB-LP	AT-LB	Competitive	19	11	4	12	1
		AT-LP	Competitive	19	14	4	5	0
		LB-LP	Competitive	10	18	10	5	0
4	AF-AP-AT-LB	AF-AP	Competitive	2	21	0	0	5
		AF-AT	Parasitic	5	18	3	1	5
		AF-LB	Competitive	21	10	6	10	1
		AP-AT	Parasitic	4	19	2	2	5
		AP-LB	Competitive	21	10	6	10	1
		AT-LB	Parasitic	17	11	8	12	1
	AF-AP-AT-LP	AF-AP	Competitive	0	19	0	0	5
		AF-AT	Parasitic	3	17	0	4	5
		AF-LP	Competitive	15	12	4	6	1
		AP-AT	Parasitic	3	17	0	4	5
		AP-LP	Competitive	15	12	4	6	1
		AT-LP	Parasitic	13	12	5	7	2
	AF-AP-LB-LP	AF-AP	Competitive	1	21	0	0	7
		AF-LB	Competitive	21	11	4	12	2
		AF-LP	Competitive	22	12	4	6	1
		AP-LB	Competitive	20	11	4	12	2
		AP-LP	Competitive	22	12	3	7	1
		LB-LP	Competitive	6	19	9	9	0
	AF-AT-LB-LP	AF-AT	Parasitic	5	18	3	1	5
		AF-LB	Competitive	22	10	5	10	1
		AF-LP	Competitive	21	12	3	6	2
		AT-LB	Parasitic	19	11	6	13	1
		AT-LP	Parasitic	15	14	5	8	2
		LB-LP	Competitive	6	20	5	7	4
	AP-AT-LB-LP	AP-AT	Parasitic	5	18	1	1	5
		AP-LB	Competitive	20	10	5	11	1
		AP-LP	Competitive	20	12	2	8	1
		AT-LB	Parasitic	18	11	7	11	1
		AT-LP	Parasitic	16	14	4	6	2
		LB-LP	Competitive	6	20	5	7	4
5	AF-AP-AT-LB-LP	AF-AP	Competitive	3	20	0	1	5
		AF-AT	Parasitic	5	18	3	1	5
		AF-LB	Competitive	22	10	5	11	1
		AF-LP	Competitive	21	12	2	6	2
		AP-AT	Parasitic	4	19	1	2	6
		AP-LB	Competitive	21	10	5	12	1
		AP-LP	Competitive	18	13	2	7	2
		AT-LB	Parasitic	18	11	7	13	1
		AT-LP	Parasitic	15	14	4	8	2
		LB-LP	Competitive	6	20	4	8	4

AF-*Acetobacter fabarum*; AP-*Acetobacter pomorum*; AT-*Acetobacter tropicalis*; LB-*Lactobacillus brevis*; LP-*Lactobacillus plantarum*

Table S3C. Predicted community metabolite use patterns for competitive, parasitic and mutualistic interactions - minimal medium.

Community size	Community composition	Pairwise comparison	Interaction Type	Single-use	Co-consumed	Cross-fed	Single-produced	Co-produced
2	AF_AP	AF-AP	Parasitic	0	5	10	0	1
	AF_AT	AF-AT	Parasitic	1	4	12	1	1
	AF_LP	AF-LP	Mutualistic	2	3	19	2	1
	AP_AT	AP-AT	Competitive	1	4	12	1	1
	AP_LP	AP-LP	Parasitic	2	3	16	2	1
	AT_LP	AT-LP	Parasitic	2	3	19	2	1
3	AF-AP-AT	AF-AP	Parasitic	6	14	0	1	1
		AF-AT	Parasitic	2	4	13	3	1
		AP-AT	Competitive	1	4	12	5	1
	AF-AP-LP	AF-AP	Parasitic	2	10	5	7	3
		AF-LP	Mutualistic	7	4	14	2	1
		AP-LP	Parasitic	4	5	12	4	2
	AF-AT-LP	AF-AT	Parasitic	2	10	4	8	7
		AF-LP	Mutualistic	10	4	13	5	0
		AT-LP	Parasitic	4	3	19	5	1
	AF-LB-LP	AF-LB	Mutualistic	14	6	15	6	1
		AF-LP	Mutualistic	2	5	17	15	3
		LB-LP	Mutualistic	9	8	17	6	0
	AP-AT-LP	AP-AT	Competitive	2	10	3	6	7
		AP-LP	Parasitic	8	4	12	3	2
		AT-LP	Parasitic	3	3	18	4	1
	AP-LB-LP	AP-LB	Parasitic	14	5	15	6	1
		AP-LP	Parasitic	2	5	14	17	2
		LB-LP	Mutualistic	8	9	17	3	1
	AT-LB-LP	AT-LB	Parasitic	10	8	15	10	0
		AT-LP	Parasitic	6	5	15	13	3
		LB-LP	Mutualistic	8	9	20	3	0
4	AF-AP-AT-LB	AP-AT	Competitive	0	0	0	0	0
	AF-AP-AT-LP	AF-AP	Parasitic	1	12	2	7	3
		AF-AT	Parasitic	3	10	2	6	5
		AF-LP	Mutualistic	9	4	13	2	1
		AP-AT	Competitive	2	9	4	7	4
		AP-LP	Parasitic	8	5	11	3	2
		AT-LP	Parasitic	7	3	14	5	1
	AF-AP-LB-LP	AF-AP	Parasitic	1	11	1	14	3
		AF-LB	Mutualistic	21	5	8	7	0
		AF-LP	Mutualistic	8	5	9	15	2
		AP-LB	Parasitic	18	6	9	5	1
		AP-LP	Parasitic	4	6	11	14	2
		LB-LP	Mutualistic	8	8	18	5	0
	AF-AT-LB-LP	AF-AT	Parasitic	3	9	5	10	7
		AF-LB	Mutualistic	20	8	6	10	0
		AF-LP	Mutualistic	12	6	10	14	1
		AT-LB	Parasitic	12	7	12	13	1
		AT-LP	Parasitic	7	3	17	16	2
		LB-LP	Mutualistic	10	8	20	4	0
	AP-AT-LB-LP	AP-AT	Competitive	2	11	0	12	6
		AP-LB	Parasitic	18	7	8	7	1
		AP-LP	Parasitic	8	5	11	14	2
		AT-LB	Parasitic	19	6	9	8	1
		AT-LP	Parasitic	7	5	12	15	2
		LB-LP	Mutualistic	9	8	20	4	0
5	AF-AP-AT-LB-LP	AF-AP	Parasitic	4	11	0	9	7
		AF-AT	Parasitic	2	11	2	6	6

AF-LB	Mutualistic	23	7	5	9	1
AF-LP	Mutualistic	10	6	10	14	2
AP-AT	Competitive	2	11	0	11	6
AP-LB	Parasitic	17	7	9	8	1
AP-LP	Parasitic	8	5	12	15	2
AT-LB	Parasitic	23	6	5	9	1
AT-LP	Parasitic	9	5	11	13	2
LB-LP	Mutualistic	10	8	20	4	0

AF-*Acetobacter fabarum*; AP-*Acetobacter pomorum*; AT-*Acetobacter tropicalis*; LB-*Lactobacillus brevis*; LP-*Lactobacillus plantarum*

Table S3D. Summary statistics for Figure 3.

	f.value	p.value	Tukey comparison	p.value
**Fig 3A (Rich competitive)**	84.7	<2e-16	co_produced-co_consumed	0
			cross_fed-co_consumed	0
			single_produced-co_consumed	0
			single_use-co_consumed	0.0016512
			cross_fed-co_produced	0.9989412
			single_produced-co_produced	0.5057208
			single_use-co_produced	0
			single_produced-cross_fed	0.6749947
			single_use-cross_fed	0
			single_use-single_produced	0
**Fig 3B (Base competitive)**	58.56	<2e-16	co_produced-co_consumed	0
			cross_fed-co_consumed	0
			single_produced-co_consumed	0
			single_use-co_consumed	0.0008057
			cross_fed-co_produced	0.6838525
			single_produced-co_produced	0.0444179
			single_use-co_produced	0
			single_produced-cross_fed	0.5763813
			single_use-cross_fed	0
			single_use-single_produced	0.0000001
**Fig 3C (Minimal competitive)**	2.576	0.0622	co_produced-co_consumed	0.3776919
			cross_fed-co_consumed	0.9309045
			single_produced-co_consumed	0.9730605
			single_use-co_consumed	0.0576864
			cross_fed-co_produced	0.8330905
			single_produced-co_produced	0.7349723
			single_use-co_produced	0.8358828
			single_produced-cross_fed	0.9997113
			single_use-cross_fed	0.2598555
			single_use-single_produced	0.1893655
**Fig 3D (Rich parasitic)**	134.2	<2e-16	co_produced-co_consumed	0
			cross_fed-co_consumed	0
			single_produced-co_consumed	0
			single_use-co_consumed	0
			cross_fed-co_produced	0.9966574
			single_produced-co_produced	0.186073
			single_use-co_produced	0
			single_produced-cross_fed	0.3522236
			single_use-cross_fed	0
			single_use-single_produced	0
**Fig 3E (Base parasitic)**	50.27	<2e-16	co_produced-co_consumed	0
			cross_fed-co_consumed	0
			single_produced-co_consumed	0
			single_use-co_consumed	0.0001462

			cross_fed-co_produced	0.9879244
			single_produced-co_produced	0.129792
			single_use-co_produced	0
			single_produced-cross_fed	0.3350138
			single_use-cross_fed	0
			single_use-single_produced	0.0000259
**Fig 3F (Minimal parasitic)**	14.67	2.37E-10	co_produced-co_consumed	0.0002978
			cross_fed-co_consumed	0.0107213
			single_produced-co_consumed	0.9965538
			single_use-co_consumed	0.9566379
			cross_fed-co_produced	0
			single_produced-co_produced	0.0000684
			single_use-co_produced	0.0040358
			single_produced-cross_fed	0.0312083
			single_use-cross_fed	0.00094
			single_use-single_produced	0.8297301
**Fig 3G (Minimal mutualisitic)**	33.38	<2e-16	co_produced-co_consumed	0.0003301
			cross_fed-co_consumed	0
			single_produced-co_consumed	0.999416
			single_use-co_consumed	0.0177718
			cross_fed-co_produced	0
			single_produced-co_produced	0.000141
			single_use-co_produced	0
			single_produced-cross_fed	0.0000001
			single_use-cross_fed	0.0066907
			single_use-single_produced	0.0334452
