# Supplementary material for: Predicted Metabolic Function of the Gut Microbiota of Drosophila melanogaster
Source: mSystems. 2021 May 4;6(3):e01369-20. doi: 10.1128/mSystems.01369-20 (PMC8269265; doi:10.1128/mSystems.01369-20)
Supplement: TABLE S4 [file msystems.01369-20-st004.pdf]

Table S4A. Metabolite use pattern - rich medium.

Present in medium	Metabolites	Number of times metabolite is used: Single-use	Co-consumed	Cross-fed	Single-produced	Co-produced	Total number of times metabolite is used in all simulations	Frequency of metabolite use: Single-use	Co-consumed	Cross-fed	Single-produced	Co-produced
Yes	meso-2,6-Diaminoheptanedioate	32	0	0	0	0	32	1.0	0.0	0.0	0.0	0.0
Yes	D-Alanine	0	0	0	48	24	72	0.0	0.0	0.0	0.7	0.3
Yes	Alanine	46	7	0	0	0	53	0.9	0.1	0.0	0.0	0.0
Yes	Arginine	0	80	0	0	0	80	0.0	1.0	0.0	0.0	0.0
Yes	Asparagine	0	80	0	0	0	80	0.0	1.0	0.0	0.0	0.0
Yes	Aspartate	45	27	0	0	0	72	0.6	0.4	0.0	0.0	0.0
Yes	Cysteine	32	0	0	0	0	32	1.0	0.0	0.0	0.0	0.0
Yes	Glutamine	7	73	0	0	0	80	0.1	0.9	0.0	0.0	0.0
Yes	Glutamate	33	2	1	0	0	36	0.9	0.1	0.0	0.0	0.0
Yes	Glycine	48	24	0	0	0	72	0.7	0.3	0.0	0.0	0.0
Yes	Homocysteine	0	0	0	32	0	32	0.0	0.0	0.0	1.0	0.0
Yes	Histidine	32	0	0	0	0	32	1.0	0.0	0.0	0.0	0.0
Yes	Isoleucine	48	8	0	0	0	56	0.9	0.1	0.0	0.0	0.0
Yes	Leucine	49	7	0	0	0	56	0.9	0.1	0.0	0.0	0.0
Yes	Lysine	48	8	0	0	0	56	0.9	0.1	0.0	0.0	0.0
Yes	Methionine	15	0	6	26	7	54	0.3	0.0	0.1	0.5	0.1
Yes	Ornithine	0	0	0	48	25	73	0.0	0.0	0.0	0.7	0.3
Yes	Phenylalanine	0	80	0	0	0	80	0.0	1.0	0.0	0.0	0.0
Yes	Proline	1	79	0	0	0	80	0.0	1.0	0.0	0.0	0.0
Yes	Serine	0	48	32	0	0	80	0.0	0.6	0.4	0.0	0.0
Yes	Threonine	48	8	0	0	0	56	0.9	0.1	0.0	0.0	0.0
Yes	Tryptophan	0	80	0	0	0	80	0.0	1.0	0.0	0.0	0.0
Yes	Tyrosine	1	79	0	0	0	80	0.0	1.0	0.0	0.0	0.0
Yes	Valine	48	8	0	0	0	56	0.9	0.1	0.0	0.0	0.0
Yes	(2-Aminoethyl)phosphonate	32	0	0	0	0	32	1.0	0.0	0.0	0.0	0.0
Yes	2-Dehydro-3-deoxy-D-gluconate	1	0	0	0	0	1	1.0	0.0	0.0	0.0	0.0
Yes	L-2-hydroxyisocaproate	1	0	0	0	0	1	1.0	0.0	0.0	0.0	0.0
Yes	(R)-3-(4-Hydroxyphenyl)lactate	1	0	0	0	0	1	1.0	0.0	0.0	0.0	0.0
Yes	4-Aminobutanoate	47	6	0	0	0	53	0.9	0.1	0.0	0.0	0.0
Yes	Acetate	5	12	29	19	8	73	0.1	0.2	0.4	0.3	0.1
Yes	Acetaldehyde	50	6	0	0	0	56	0.9	0.1	0.0	0.0	0.0
Yes	N-Acetyl-D-glucosamine	50	22	0	0	0	72	0.7	0.3	0.0	0.0	0.0
Yes	R Acetoin	24	11	24	15	0	74	0.3	0.1	0.3	0.2	0.0
Yes	S Acetoin	46	2	0	0	0	48	1.0	0.0	0.0	0.0	0.0

Yes	2-Oxoglutarate	5	3	28	20	24	80	0.1	0.0	0.4	0.3	0.3
Yes	(R,R)-2,3-Butanediol	39	0	0	0	0	39	1.0	0.0	0.0	0.0	0.0
Yes	(S,S)-2,3-Butanediol	45	9	0	0	0	54	0.8	0.2	0.0	0.0	0.0
Yes	Citrate	2	0	0	0	0	2	1.0	0.0	0.0	0.0	0.0
Yes	Ethanol	30	0	0	0	0	30	1.0	0.0	0.0	0.0	0.0
Yes	Formaldehyde						0					
Yes	Formate	0	0	0	19	2	21	0.0	0.0	0.0	0.9	0.1
Yes	D-Fructose	25	0	0	0	0	25	1.0	0.0	0.0	0.0	0.0
Yes	Fumarate	1	0	0	0	0	1	1.0	0.0	0.0	0.0	0.0
Yes	D-Glucose	18	7	0	0	0	25	0.7	0.3	0.0	0.0	0.0
Yes	D-Gluconate	6	0	0	0	0	6	1.0	0.0	0.0	0.0	0.0
Yes	Glycerol	46	15	4	3	0	68	0.7	0.2	0.1	0.0	0.0
Yes	Glycerol 3-phosphate	48	4	0	0	0	52	0.9	0.1	0.0	0.0	0.0
Yes	Glycolate						0					
Yes	Imidazole lactate	32	0	0	0	0	32	1.0	0.0	0.0	0.0	0.0
Yes	D-Lactate	0	0	0	6	0	6	0.0	0.0	0.0	1.0	0.0
Yes	L-Lactate	0	0	0	6	0	6	0.0	0.0	0.0	1.0	0.0
Yes	Malate	1	0	0	37	12	50	0.0	0.0	0.0	0.7	0.2
Yes	Maltose	42	6	0	0	0	48	0.9	0.1	0.0	0.0	0.0
Yes	Maltotetraose	32	0	0	0	0	32	1.0	0.0	0.0	0.0	0.0
Yes	D-Mannose	20	0	0	0	0	20	1.0	0.0	0.0	0.0	0.0
Yes	D-Mannitol	23	0	0	0	0	23	1.0	0.0	0.0	0.0	0.0
Yes	Methylglyoxal	35	2	0	0	0	37	0.9	0.1	0.0	0.0	0.0
Yes	Pyruvate						0					
Yes	D-Sorbitol	14	0	0	0	0	14	1.0	0.0	0.0	0.0	0.0
Yes	Succinate	13	0	19	24	6	62	0.2	0.0	0.3	0.4	0.1
Yes	Succinyl-CoA	32	48	0	0	0	80	0.4	0.6	0.0	0.0	0.0
Yes	Sucrose	51	9	0	0	0	60	0.9	0.2	0.0	0.0	0.0
Yes	Trehalose	49	7	0	0	0	56	0.9	0.1	0.0	0.0	0.0
Yes	Adenine	48	8	0	0	0	56	0.9	0.1	0.0	0.0	0.0
Yes	Adenosine	32	0	0	0	0	32	1.0	0.0	0.0	0.0	0.0
Yes	Cytosine	1	0	0	0	0	1	1.0	0.0	0.0	0.0	0.0
Yes	Cytidine	32	0	0	0	0	32	1.0	0.0	0.0	0.0	0.0
Yes	Deoxyadenosine	26	0	0	0	0	26	1.0	0.0	0.0	0.0	0.0
Yes	Deoxycytidine	40	1	0	0	0	41	1.0	0.0	0.0	0.0	0.0
Yes	Deoxyribose	3	0	0	0	0	3	1.0	0.0	0.0	0.0	0.0
No	dUMP	12	19	39	7	0	77	0.2	0.2	0.5	0.1	0.0
Yes	Deoxyuridine	29	1	14	20	0	64	0.5	0.0	0.2	0.3	0.0
Yes	Guanine	48	8	0	0	0	56	0.9	0.1	0.0	0.0	0.0
Yes	Hypoxanthine	19	6	12	5	0	42	0.5	0.1	0.3	0.1	0.0
Yes	Inosine	48	0	0	0	0	48	1.0	0.0	0.0	0.0	0.0

Yes	Orotate						0					
Yes	Thymidine	48	8	0	0	0	56	0.9	0.1	0.0	0.0	0.0
Yes	Uracil	4	20	46	2	8	80	0.1	0.3	0.6	0.0	0.1
Yes	Uridine	45	8	3	0	0	56	0.8	0.1	0.1	0.0	0.0
Yes	Xanthine	8	2	9	23	0	42	0.2	0.0	0.2	0.5	0.0
Yes	Biotin (B7)	0	80	0	0	0	80	0.0	1.0	0.0	0.0	0.0
Yes	Coenzyme A	24	0	8	24	0	56	0.4	0.0	0.1	0.4	0.0
Yes	Dihydropteroate						0					
Yes	1-deoxy-D-xylulose 5-phosphate	32	0	0	0	0	32	1.0	0.0	0.0	0.0	0.0
Yes	Folate (B9)	48	8	0	0	0	56	0.9	0.1	0.0	0.0	0.0
Yes	Nicotinate						0					
Yes	Nicotinamide D-ribonucleotide	0	80	0	0	0	80	0.0	1.0	0.0	0.0	0.0
Yes	Pyridoxine 5-phosphate (B6)	48	24	0	0	0	72	0.7	0.3	0.0	0.0	0.0
Yes	Pantothenate (B5)						0					
Yes	Pyridoxamine (B6)	0	0	0	44	0	44	0.0	0.0	0.0	1.0	0.0
Yes	Pyridoxal 5'-phosphate (B6)	48	8	0	0	0	56	0.9	0.1	0.0	0.0	0.0
Yes	Riboflavin (B2)	48	24	0	0	0	72	0.7	0.3	0.0	0.0	0.0
Yes	Tetrahydrofolate (B9)	48	24	0	0	0	72	0.7	0.3	0.0	0.0	0.0
Yes	Thiamin (B1)	24	0	8	24	0	56	0.4	0.0	0.1	0.4	0.0
No	Toxopyrimidine	32	0	0	0	0	32	1.0	0.0	0.0	0.0	0.0
Yes	Ammonium	0	0	0	23	57	80	0.0	0.0	0.0	0.3	0.7
No	L-Cysteinylglycine	49	7	0	0	0	56	0.9	0.1	0.0	0.0	0.0
Yes	L-methionyl-L-alanine	2	78	0	0	0	80	0.0	1.0	0.0	0.0	0.0
Yes	Hydrogen sulfide	48	24	0	0	0	72	0.7	0.3	0.0	0.0	0.0
Yes	Sulfate	48	24	0	0	0	72	0.7	0.3	0.0	0.0	0.0

Table S4B. Metabolite use pattern - base medium.

		**Number of times metabolite is used**						**Frequency of metabolite use**				
**Present in medium**	**Metabolites**	**Single-use**	**Co-consumed**	**Cross-fed**	**Single-produced**	**Co-produced**	**Total number of times metabolite is used in all simulations**	**Single-use**	**Co-consumed**	**Cross-fed**	**Single-produced**	**Co-produced**
Yes	meso-2,6-Diaminoheptanedioate	32	0	0	0	0	32	1.0	0.0	0.0	0.0	0.0
No	D-Alanine	0	0	0	48	24	72	0.0	0.0	0.0	0.7	0.3
Yes	Alanine	48	7	0	0	0	55	0.9	0.1	0.0	0.0	0.0
Yes	Arginine	0	80	0	0	0	80	0.0	1.0	0.0	0.0	0.0
Yes	Asparagine	0	80	0	0	0	80	0.0	1.0	0.0	0.0	0.0
Yes	Aspartate	37	20	0	0	0	57	0.6	0.4	0.0	0.0	0.0
Yes	Cysteine	48	8	0	0	0	56	0.9	0.1	0.0	0.0	0.0

Yes	Glutamine	0	80	0	0	0	80	0.0	1.0	0.0	0.0	0.0
Yes	Glutamate	5	0	5	5	0	15	0.3	0.0	0.3	0.3	0.0
Yes	Glycine	18	18	34	3	1	74	0.2	0.2	0.5	0.0	0.0
No	Homocysteine	0	0	0	32	0	32	0.0	0.0	0.0	1.0	0.0
Yes	Histidine	32	0	0	0	0	32	1.0	0.0	0.0	0.0	0.0
Yes	Isoleucine	48	8	0	0	0	56	0.9	0.1	0.0	0.0	0.0
Yes	Leucine	48	8	0	0	0	56	0.9	0.1	0.0	0.0	0.0
Yes	Lysine	48	8	0	0	0	56	0.9	0.1	0.0	0.0	0.0
Yes	Methionine	0	80	0	0	0	80	0.0	1.0	0.0	0.0	0.0
No	Ornithine	0	0	0	37	38	75	0.0	0.0	0.0	0.5	0.5
Yes	Phenylalanine	0	80	0	0	0	80	0.0	1.0	0.0	0.0	0.0
Yes	Proline	0	80	0	0	0	80	0.0	1.0	0.0	0.0	0.0
Yes	Serine	0	54	26	0	0	80	0.0	0.7	0.3	0.0	0.0
Yes	Threonine	48	8	0	0	0	56	0.9	0.1	0.0	0.0	0.0
Yes	Tryptophan	0	80	0	0	0	80	0.0	1.0	0.0	0.0	0.0
Yes	Tyrosine	0	80	0	0	0	80	0.0	1.0	0.0	0.0	0.0
Yes	Valine	48	8	0	0	0	56	0.9	0.1	0.0	0.0	0.0
No	(2-Aminoethyl)phosphonate						0					
No	2-Dehydro-3-deoxy-D-gluconate						0					
No	L-2-hydroxyisocaproate						0					
No	(R)-3-(4-Hydroxyphenyl)lactate						0					
No	4-Aminobutanoate						0					
No	Acetate	5	19	47	5	3	79	0.1	0.2	0.6	0.1	0.0
No	Acetaldehyde	13	0	11	16	2	42	0.3	0.0	0.3	0.4	0.0
No	N-Acetyl-D-glucosamine						0					
No	R Acetoin	17	4	25	19	3	68	0.3	0.1	0.4	0.3	0.0
No	S Acetoin						0					
No	2-Oxoglutarate	1	0	6	33	39	79	0.0	0.0	0.1	0.4	0.5
No	(R,R)-2,3-Butanediol						0					
No	(S,S)-2,3-Butanediol						0					
No	Citrate						0					
No	Ethanol						0					
No	Formaldehyde						0					
No	Formate	0	0	0	42	31	73	0.0	0.0	0.0	0.6	0.4
No	D-Fructose						0					
No	Fumarate						0					
Yes	D-Glucose	0	80	0	0	0	80	0.0	1.0	0.0	0.0	0.0
No	D-Gluconate						0					
Yes	Glycerol	46	19	0	0	0	65	0.7	0.3	0.0	0.0	0.0
No	Glycerol 3-phosphate						0					
No	Glycolate	0	0	0	39	23	62	0.0	0.0	0.0	0.6	0.4

No	Imidazole lactate	12	0	8	12	0	32	0.4	0.0	0.3	0.4	0.0
No	D-Lactate						0					
No	L-Lactate						0					
No	Malate	0	0	0	17	3	20	0.0	0.0	0.0	0.9	0.2
No	Maltose						0					
No	Maltotetraose						0					
No	D-Mannose						0					
No	D-Mannitol						0					
No	Methylglyoxal						0					
No	Pyruvate						0					
No	D-Sorbitol						0					
No	Succinate	4	0	16	4	7	31	0.1	0.0	0.5	0.1	0.2
No	Succinyl-CoA	17	3	19	14	0	53	0.3	0.1	0.4	0.3	0.0
No	Sucrose						0					
No	Trehalose						0					
No	Adenine	5	0	5	5	0	15	0.3	0.0	0.3	0.3	0.0
No	Adenosine						0					
No	Cytosine						0					
No	Cytidine						0					
No	Deoxyadenosine						0					
No	Deoxycytidine						0					
No	Deoxyribose						0					
No	dUMP	11	13	26	6	0	56	0.2	0.2	0.5	0.1	0.0
No	Deoxyuridine						0					
No	Guanine						0					
No	Hypoxanthine						0					
No	Inosine						0					
No	Orotate	0	24	32	0	0	56	0.0	0.4	0.6	0.0	0.0
No	Thymidine						0					
No	Uracil						0					
No	Uridine						0					
No	Xanthine						0					
Yes	Biotin (B7)	0	80	0	0	0	80	0.0	1.0	0.0	0.0	0.0
No	Coenzyme A	5	0	5	5	0	15	0.3	0.0	0.3	0.3	0.0
Yes	Dihydropteroate	32	0	0	0	0	32	1.0	0.0	0.0	0.0	0.0
Yes	1-deoxy-D-xylulose 5-phosphate	32	0	0	0	0	32	1.0	0.0	0.0	0.0	0.0
No	Folate (B9)	12	0	8	12	0	32	0.4	0.0	0.3	0.4	0.0
Yes	Nicotinate	48	8	0	0	0	56	0.9	0.1	0.0	0.0	0.0
No	Nicotinamide D-ribonucleotide	12	18	36	7	0	73	0.2	0.2	0.5	0.1	0.0
No	Pyridoxine 5-phosphate (B6)	0	1	4	0	0	5	0.0	0.2	0.8	0.0	0.0
Yes	Pantothenate (B5)	46	31	0	0	0	77	0.6	0.4	0.0	0.0	0.0

Yes	Pyridoxamine (B6)	0	0	0	48	3	51	0.0	0.0	0.0	0.9	0.1
Yes	Pyridoxal 5'-phosphate (B6)	48	8	0	0	0	56	0.9	0.1	0.0	0.0	0.0
No	Riboflavin (B2)	8	1	8	8	0	25	0.3	0.0	0.3	0.3	0.0
No	Tetrahydrofolate (B9)	9	2	10	8	0	29	0.3	0.1	0.3	0.3	0.0
No	Thiamin (B1)	9	0	7	25	0	41	0.2	0.0	0.2	0.6	0.0
No	Toxopyrimidine	32	0	0	0	0	32	1.0	0.0	0.0	0.0	0.0
Yes	Ammonium	0	0	0	48	24	72	0.0	0.0	0.0	0.7	0.3
No	L-Cysteinylglycine						0					
No	L-methionyl-L-alanine						0					
Yes	Hydrogen sulfide	48	24	0	0	0	72	0.7	0.3	0.0	0.0	0.0
Yes	Sulfate	48	23	0	0	0	71	0.7	0.3	0.0	0.0	0.0

Table S4C. Metabolite use pattern - minimal medium.

		Number of times metabolite is used						Frequency of metabolite use				
Present in medium	Metabolite name	Single-use	Co-consumed	Cross-fed	Single-produced	Co-produced	Total number of times metabolite is used in all simulations	Single -use	Co-consumed	Cross -fed	Single-produced	Co-produced
No	meso-2,6-Diaminoheptanedioate	12	0	7	12	0	31	0.4	0.0	0.2	0.4	0.0
No	D-Alanine	0	0	0	36	18	54	0.0	0.0	0.0	0.7	0.3
No	Alanine						0					
No	Arginine	2	21	34	2	1	60	0.0	0.4	0.6	0.0	0.0
No	Asparagine	13	8	22	10	0	53	0.2	0.2	0.4	0.2	0.0
No	Aspartate						0					
No	Cysteine	12	0	7	12	0	31	0.4	0.0	0.2	0.4	0.0
No	Glutamine	1	0	1	1	0	3	0.3	0.0	0.3	0.3	0.0
No	Glutamate	11	0	8	11	1	31	0.4	0.0	0.3	0.4	0.0
No	Glycine	14	7	18	9	0	48	0.3	0.1	0.4	0.2	0.0
No	Homocysteine	0	0	0	19	0	19	0.0	0.0	0.0	1.0	0.0
No	Histidine	11	0	8	12	1	32	0.3	0.0	0.3	0.4	0.0
No	Isoleucine	8	7	28	4	7	54	0.1	0.1	0.5	0.1	0.1
No	Leucine	11	7	25	7	3	53	0.2	0.1	0.5	0.1	0.1
No	Lysine	12	0	7	12	0	31	0.4	0.0	0.2	0.4	0.0
No	Methionine	12	0	7	12	0	31	0.4	0.0	0.2	0.4	0.0
No	Ornithine	2	1	34	2	21	60	0.0	0.0	0.6	0.0	0.4
No	Phenylalanine	0	25	35	0	0	60	0.0	0.4	0.6	0.0	0.0
No	Proline	3	18	24	3	1	49	0.1	0.4	0.5	0.1	0.0
No	Serine	0	24	31	0	0	55	0.0	0.4	0.6	0.0	0.0
No	Threonine	12	0	7	12	0	31	0.4	0.0	0.2	0.4	0.0
No	Tryptophan	13	6	25	11	1	56	0.2	0.1	0.4	0.2	0.0

No	Tyrosine	3	21	31	3	0	58	0.1	0.4	0.5	0.1	0.0
No	Valine	13	7	23	9	1	53	0.2	0.1	0.4	0.2	0.0
No	(2-Aminoethyl)phosphonate						0					
No	2-Dehydro-3-deoxy-D-gluconate						0					
No	L-2-hydroxyisocaproate						0					
No	(R)-3-(4-Hydroxyphenyl)lactate	7	0	3	7	0	17	0.4	0.0	0.2	0.4	0.0
No	4-Aminobutanoate						0					
No	Acetate	12	13	27	7	0	59	0.2	0.2	0.5	0.1	0.0
No	Acetaldehyde	2	0	3	2	0	7	0.3	0.0	0.4	0.3	0.0
No	N-Acetyl-D-glucosamine						0					
No	R Acetoin	5	0	9	5	1	20	0.3	0.0	0.5	0.3	0.1
No	S Acetoin						0					
No	2-Oxoglutarate	6	0	29	9	16	60	0.1	0.0	0.5	0.2	0.3
No	(R,R)-2,3-Butanediol						0					
No	(S,S)-2,3-Butanediol						0					
No	Citrate						0					
No	Ethanol						0					
No	Formaldehyde	0	0	0	34	6	40	0.0	0.0	0.0	0.9	0.2
No	Formate	12	0	8	12	0	32	0.4	0.0	0.3	0.4	0.0
No	D-Fructose						0					
No	Fumarate						0					
Yes	D-Glucose	0	61	0	0	0	61	0.0	1.0	0.0	0.0	0.0
No	D-Gluconate						0					
Yes	Glycerol	24	36	0	0	0	60	0.4	0.6	0.0	0.0	0.0
No	Glycerol 3-phosphate						0					
No	Glycolate	0	0	0	26	32	58	0.0	0.0	0.0	0.4	0.6
No	Imidazole lactate	8	0	4	8	0	20	0.4	0.0	0.2	0.4	0.0
No	D-Lactate						0					
No	L-Lactate						0					
No	Malate	4	0	5	5	2	16	0.3	0.0	0.3	0.3	0.1
No	Maltose						0					
No	Maltotetraose						0					
No	D-Mannose						0					
No	D-Mannitol						0					
No	Methylglyoxal						0					
No	Pyruvate	11	0	6	11	0	28	0.4	0.0	0.2	0.4	0.0
No	D-Sorbitol						0					
No	Succinate	10	0	7	12	1	30	0.3	0.0	0.2	0.4	0.0
No	Succinyl-CoA	16	5	23	13	0	57	0.3	0.1	0.4	0.2	0.0
No	Sucrose						0					

No	Trehalose						0					
No	Adenine	12	0	7	12	0	31	0.4	0.0	0.2	0.4	0.0
No	Adenosine						0					
No	Cytosine						0					
No	Cytidine						0					
No	Deoxyadenosine						0					
No	Deoxycytidine						0					
No	Deoxyribose						0					
No	dUMP	6	16	26	6	0	54	0.1	0.3	0.5	0.1	0.0
No	Deoxyuridine						0					
No	Guanine						0					
No	Hypoxanthine						0					
No	Inosine						0					
No	Orotate	5	2	8	4	0	19	0.3	0.1	0.4	0.2	0.0
No	Thymidine						0					
No	Uracil						0					
No	Uridine						0					
No	Xanthine						0					
No	Biotin (B7)	8	21	26	5	0	60	0.1	0.4	0.4	0.1	0.0
No	Coenzyme A	12	0	7	12	0	31	0.4	0.0	0.2	0.4	0.0
No	Dihydropteroate						0					
No	1-deoxy-D-xylulose 5-phosphate						0					
No	Folate (B9)	12	0	7	12	0	31	0.4	0.0	0.2	0.4	0.0
No	Nicotinate						0					
No	Nicotinamide D-ribonucleotide	15	8	25	10	0	58	0.3	0.1	0.4	0.2	0.0
No	Pyridoxine 5-phosphate (B6)	0	1	5	0	0	6	0.0	0.2	0.8	0.0	0.0
No	Pantothenate (B5)						0					
No	Pyridoxamine (B6)	2	1	0	0	0	3	0.7	0.3	0.0	0.0	0.0
Yes	Pyridoxal 5'-phosphate (B6)	30	3	2	4	0	39	0.8	0.1	0.1	0.1	0.0
No	Riboflavin (B2)	10	2	12	9	0	33	0.3	0.1	0.4	0.3	0.0
No	Tetrahydrofolate (B9)	3	1	7	3	0	14	0.2	0.1	0.5	0.2	0.0
No	Thiamin (B1)	12	0	7	12	0	31	0.4	0.0	0.2	0.4	0.0
No	Toxopyrimidine						0					
Yes	Ammonium	0	61	0	0	0	61	0.0	1.0	0.0	0.0	0.0
No	L-Cysteinylglycine						0					
No	L-methionyl-L-alanine						0					
No	Hydrogen sulfide	12	0	19	16	6	53	0.2	0.0	0.4	0.3	0.1
Yes	Sulfate	36	18	0	0	0	54	0.7	0.3	0.0	0.0	0.0

Table S4D. Effect of community size and medium type on metabolite richness. Tests with significant p values are shown in bold.

	Consumption		Production	
**ANOVA**	Effect test	P-value	Effect test	P-value
Community size	$F_{4,81.63} = 0.416$	0.7968	$F_{4,97.96} = 0.227$	0.9229
Medium type	**$F_{2,196.72} = 42.572$**	**4.3×10^{-16}**	**$F_{2,197.55} = 3.397$**	**0.0354**
Interaction	**$F_{8,196.72} = 3.803$**	**0.0004**	**$F_{8,197.55} = 2.404$**	**0.0170**
**Analysis of deviance**	Effect test	P-value	Effect test	P-value
Microbial taxa	**$\chi^2_1 = 85.86$**	**$< 2.2 \times 10^{-16}$**	**$\chi^2_1 = 7.89$**	**0.0050**
Microbial treatment	$\chi^2_1 = 2.437$	0.1185	$\chi^2_1 = 0.352$	0.5529
**R^2**				
Marginal	0.5734709		0.1072632	
Conditional	0.760849		0.207347	
