# Supplementary material for: Predicted Metabolic Function of the Gut Microbiota of Drosophila melanogaster
Source: mSystems. 2021 May 4;6(3):e01369-20. doi: 10.1128/mSystems.01369-20 (PMC8269265; doi:10.1128/mSystems.01369-20)
Supplement: TABLE S5 [file msystems.01369-20-st005.pdf]

Table S5A. Predicted total number of times metabolite is consumed or produced by individual bacteria in all simulations - rich medium.

	Acetobacter fabarum	*Acetobacter pomorum*	*Acetobacter tropicalis*	*Lactobacillus brevis*	*Lactobacillus plantarum*
**Total number of metabolites used in all simulations**	**41**	**40**	**52**	**65**	**64**
**Number of times used variably**	**0**	**0**	**1**	**2**	**1**
**% metabolite use variability**	**0**	**0**	**2**	**3**	**2**

	Acetobacter fabarum		*Acetobacter pomorum*		*Acetobacter tropicalis*		*Lactobacillus brevis*		*Lactobacillus plantarum*	
**Metabolite**	**Consume**	**Produce**	**Consume**	**Produce**	**Consume**	**Produce**	**Consume**	**Produce**	**Consume**	**Produce**
meso-2,6-Diaminoheptanedioate	0	0	0	0	0	0	16	0	0	0
D-Alanine	0	16	0	16	0	16	0	0	0	0
Alanine	0	0	0	0	0	0	16	0	12	0
Arginine	16	0	16	0	16	0	16	0	16	0
Asparagine	16	0	16	0	16	0	16	0	16	0
Aspartate	3	0	4	0	10	0	16	0	15	0
Cysteine	0	0	0	0	0	0	16	0	0	0
Glutamine	16	0	16	0	16	0	16	0	13	0
Glutamate	0	0	0	0	1	0	10	0	10	1
Glycine	16	0	16	0	16	0	0	0	0	0
Homocysteine	0	0	0	0	0	0	0	16	0	0
Histidine	0	0	0	0	0	0	16	0	0	0
Isoleucine	0	0	0	0	0	0	16	0	16	0
Leucine	0	0	0	0	0	0	16	0	15	0
Lysine	0	0	0	0	0	0	16	0	16	0
Methionine	0	7	0	16	0	5	8	0	1	0
Ornithine	0	16	0	16	0	16	0	2	0	1
Phenylalanine	16	0	16	0	16	0	16	0	16	0
Proline	16	0	16	0	16	0	16	0	15	0
Serine	16	0	16	0	16	0	0	16	16	0
Threonine	0	0	0	0	0	0	16	0	16	0
Tryptophan	16	0	16	0	16	0	16	0	16	0
Tyrosine	16	0	16	0	16	0	16	0	15	0
Valine	0	0	0	0	0	0	16	0	16	0
(2-Aminoethyl)phosphonate	0	0	0	0	0	0	0	0	16	0
2-Dehydro-3-deoxy-D-gluconate	0	0	0	0	0	0	2	0	0	0
L-2-hydroxyisocaproate	0	0	0	0	0	0	0	0	1	0
(R)-3-(4-Hydroxyphenyl)lactate	0	0	0	0	0	0	0	0	1	0
4-Aminobutanoate	0	0	0	0	0	0	14	0	12	0
Acetate	11	0	11	0	5	0	0	16	0	16
Acetaldehyde	8	0	12	0	8	0	4	0	5	0
N-Acetyl-D-glucosamine	16	0	0	0	0	0	16	0	14	0
R Acetoin	10	0	12	0	16	0	0	6	0	12
S Acetoin	6	0	8	0	15	0	0	0	0	0
2-Oxoglutarate	0	16	0	16	0	16	3	0	16	0
(R,R)-2,3-Butanediol	0	0	0	0	0	0	6	0	12	0
(S,S)-2,3-Butanediol	11	0	8	0	15	0	0	0	0	0
Citrate	0	0	0	0	0	0	0	0	3	0
Ethanol	0	0	0	0	6	0	0	0	7	0
Formaldehyde										
Formate	0	2	0	0	0	10	0	0	0	0
D-Fructose	0	0	0	0	0	0	2	0	12	0
Fumarate	0	0	0	0	1	0	0	0	0	0
D-Glucose	2	0	4	0	0	0	6	0	9	0
D-Gluconate	0	0	2	0	0	0	2	0	0	0
Glycerol	16	0	16	0	4	0	0	3	2	0

Glycerol 3-phosphate	0	0	0	0	0	0	13	0	15	0
Glycolate										
Imidazole lactate	0	0	0	0	0	0	0	0	16	0
D-Lactate	0	0	0	0	0	0	0	4	0	0
L-Lactate	0	0	0	0	0	0	0	3	0	0
Malate	0	14	0	14	0	6	0	0	2	0
Maltose	0	0	0	0	3	0	16	0	8	0
Maltotetraose	0	0	0	0	16	0	0	0	0	0
D-Mannose	0	0	0	0	6	0	3	0	2	0
D-Mannitol	0	0	0	0	0	0	0	0	10	0
Methylglyoxal	4	0	8	0	9	0	0	0	0	0
Pyruvate										
D-Sorbitol	0	0	0	0	0	0	2	0	7	0
Succinate	0	4	0	4	0	4	2	14	15	0
Succinyl-CoA	16	0	16	0	16	0	0	0	16	0
Sucrose	8	0	15	0	9	0	0	0	4	0
Trehalose	0	0	0	0	6	0	10	0	15	0
Adenine	0	0	0	0	0	0	16	0	16	0
Adenosine	0	0	0	0	16	0	0	0	0	0
Cytosine	0	0	0	0	0	0	0	0	2	0
Cytidine	0	0	0	0	16	0	0	0	0	0
Deoxyadenosine	0	0	0	0	10	0	0	0	0	0
Deoxycytidine	0	0	0	0	5	0	16	0	0	0
Deoxyribose	0	0	0	0	0	0	2	0	0	0
dUMP	14	0	14	0	11	3	0	14	0	6
Deoxyuridine	0	0	0	0	0	11	9	3	16	0
Guanine	0	0	0	0	0	0	16	0	16	0
Hypoxanthine	12	0	11	0	0	0	0	8	0	5
Inosine	0	0	0	0	16	0	8	0	3	0
Orotate										
Thymidine	0	0	0	0	0	0	16	0	16	0
Uracil	16	0	16	0	10	0	0	16	0	16
Uridine	0	0	0	0	0	2	16	0	16	0
Xanthine	4	0	5	0	0	16	0	0	0	0
Biotin (B7)	16	0	16	0	16	0	16	0	16	0
Coenzyme A	0	0	0	0	0	0	16	0	0	16
Dihydropteroate										
1-deoxy-D-xylulose 5-phosphate	0	0	0	0	0	0	16	0	0	0
Folate (B9)	0	0	0	0	0	0	16	0	16	0
Nicotinate										
Nicotinamide D-ribonucleotide	16	0	16	0	16	0	16	0	16	0
Pyridoxine 5-phosphate (B6)	16	0	16	0	16	0	0	0	0	0
Pantothenate (B5)										
Pyridoxamine (B6)	0	0	0	0	0	0	0	9	0	15
Pyridoxal 5'-phosphate (B6)	0	0	0	0	0	0	16	0	16	0
Riboflavin (B2)	16	0	16	0	16	0	0	0	0	0
Tetrahydrofolate (B9)	16	0	16	0	16	0	0	0	0	0
Thiamin (B1)	0	0	0	0	0	0	0	16	16	0
Toxopyrimidine	0	0	0	0	0	0	16	0	0	0
Ammonium	0	16	0	16	0	16	0	16	0	6
L-Cysteinylglycine	0	0	0	0	0	0	14	0	16	0
L-methionyl-L-alanine	16	0	16	0	16	0	15	0	15	0
Hydrogen sulfide	16	0	16	0	16	0	0	0	0	0
Sulfate	16	0	16	0	16	0	0	0	0	0

Table S5B. Predicted total number of times metabolite is consumed or produced by individual bacteria in all simulations - base medium.

	Acetobacter fabarum	*Acetobacter pomorum*	*Acetobacter tropicalis*	*Lactobacillus brevis*	*Lactobacillus plantarum*
**Total number of metabolites used in all simulations**	**35**	**35**	**35**	**47**	**43**
**Number of times used variably**	**1**	**1**	**2**	**1**	**1**
**% metabolite use variability**	**3**	**3**	**6**	**2**	**2**

**Metabolite**	**Consume**	**Produce**	**Consume**	**Produce**	**Consume**	**Produce**	**Consume**	**Produce**	**Consume**	**Produce**
meso-2,6-Diaminoheptanedioate	0	0	0	0	0	0	16	0	0	0
D-Alanine	0	16	0	16	0	16	0	0	0	0
Alanine	0	0	0	0	0	0	16	0	14	0
Arginine	16	0	16	0	16	0	16	0	16	0
Asparagine	16	0	16	0	16	0	16	0	16	0
Aspartate	6	0	8	0	12	0	2	0	12	0
Cysteine	0	0	0	0	0	0	16	0	16	0
Glutamine	16	0	16	0	16	0	16	0	16	0
Glutamate	0	0	0	0	0	0	0	5	6	0
Glycine	12	0	12	0	16	0	0	10	0	9
Homocysteine	0	0	0	0	0	0	0	16	0	0
Histidine	0	0	0	0	0	0	16	0	0	0
Isoleucine	0	0	0	0	0	0	16	0	16	0
Leucine	0	0	0	0	0	0	16	0	16	0
Lysine	0	0	0	0	0	0	16	0	16	0
Methionine	16	0	16	0	16	0	16	0	16	0
Ornithine	0	16	0	16	0	16	0	6	0	0
Phenylalanine	16	0	16	0	16	0	16	0	16	0
Proline	16	0	16	0	16	0	16	0	16	0
Serine	16	0	16	0	16	0	6	10	16	0
Threonine	0	0	0	0	0	0	16	0	16	0
Tryptophan	16	0	16	0	16	0	16	0	16	0
Tyrosine	16	0	16	0	16	0	16	0	16	0
Valine	0	0	0	0	0	0	16	0	16	0
(2-Aminoethyl)phosphonate										
2-Dehydro-3-deoxy-D-gluconate										
L-2-hydroxyisocaproate										
(R)-3-(4-Hydroxyphenyl)lactate										
4-Aminobutanoate										
Acetate	14	0	14	0	12	3	0	10	0	16
Acetaldehyde	2	0	1	0	6	0	0	5	0	6
N-Acetyl-D-glucosamine										
R Acetoin	7	0	9	0	6	2	0	7	0	12
S Acetoin										
2-Oxoglutarate	0	16	0	16	0	16	0	8	5	3
(R,R)-2,3-Butanediol										
(S,S)-2,3-Butanediol										
Citrate										
Ethanol										
Formaldehyde										
Formate	0	14	0	13	0	14	0	0	0	9
D-Fructose										
Fumarate										
D-Glucose	16	0	16	0	16	0	16	0	16	0
D-Gluconate										
Glycerol	16	0	16	0	2	0	3	0	10	0

Glycerol 3-phosphate										
Glycolate	0	10	0	12	0	16	0	0	0	8
Imidazole lactate	0	0	0	0	0	0	0	8	8	0
D-Lactate										
L-Lactate										
Malate	0	2	0	1	0	4	0	6	0	0
Maltose										
Maltotetraose										
D-Mannose										
D-Mannitol										
Methylglyoxal										
Pyruvate										
D-Sorbitol										
Succinate	0	4	0	5	0	4	0	4	10	0
Succinyl-CoA	6	1	6	2	0	13	0	0	7	0
Sucrose										
Trehalose										
Adenine	0	0	0	0	0	0	0	5	5	0
Adenosine										
Cytosine										
Cytidine										
Deoxyadenosine										
Deoxycytidine										
Deoxyribose										
dUMP	8	0	8	0	8	0	0	15	2	0
Deoxyuridine										
Guanine										
Hypoxanthine										
Inosine										
Orotate	8	0	8	0	8	0	0	15	8	0
Thymidine										
Uracil										
Uridine										
Xanthine										
Biotin (B7)	16	0	16	0	16	0	16	0	16	0
Coenzyme A	0	0	0	0	0	0	0	5	5	0
Dihydropteroate	0	0	0	0	0	0	16	0	0	0
1-deoxy-D-xylulose 5-phosphate	0	0	0	0	0	0	16	0	0	0
Folate (B9)	0	0	0	0	0	0	0	8	8	0
Nicotinate	0	0	0	0	0	0	16	0	16	0
Nicotinamide D-ribonucleotide	12	0	12	0	12	0	0	11	0	10
Pyridoxine 5-phosphate (B6)	2	0	2	0	0	3	0	0	0	0
Pantothenate (B5)	10	0	10	0	16	0	16	0	4	0
Pyridoxamine (B6)	0	0	0	0	0	0	0	11	0	16
Pyridoxal 5'-phosphate (B6)	0	0	0	0	0	0	16	0	16	0
Riboflavin (B2)	6	0	2	0	0	7	0	0	0	0
Tetrahydrofolate (B9)	6	0	4	0	0	8	0	0	0	0
Thiamin (B1)	0	0	0	0	0	0	0	16	7	0
Toxopyrimidine	0	0	0	0	0	0	16	0	0	0
Ammonium	0	16	0	16	0	16	0	0	0	1
L-Cysteinylglycine										
L-methionyl-L-alanine										
Hydrogen sulfide	16	0	16	0	16	0	0	0	0	0
Sulfate	14	0	14	0	16	0	0	0	0	0

Table S5C. Predicted total number of times metabolite is consumed or produced by individual bacteria in all simulations - minimal medium.

	Acetobacter fabarum	*Acetobacter pomorum*	*Acetobacter tropicalis*	*Lactobacillus brevis*	*Lactobacillus plantarum*
**Total number of metabolites used in all simulations**	**39**	**38**	**38**	**39**	**41**
**Number of times used variably**	**11**	**11**	**7**	**1**	**1**
**% metabolite use variability**	**28**	**29**	**18**	**3**	**2**

**Metabolite**	**Consume**	**Produce**	**Consume**	**Produce**	**Consume**	**Produce**	**Consume**	**Produce**	**Consume**	**Produce**
meso-2,6-Diaminoheptanedioate	0	0	0	0	0	0	7	0	0	7
D-Alanine	0	11	0	12	0	12	0	0	0	0
Alanine										
Arginine	4	4	10	1	4	6	0	7	14	0
Asparagine	11	0	4	1	0	5	7	0	0	9
Aspartate										
Cysteine	0	3	0	2	0	2	7	0	0	0
Glutamine	0	0	1	0	0	1	0	0	0	0
Glutamate	0	1	0	2	0	1	7	0	0	4
Glycine	2	0	4	0	5	0	7	0	0	11
Homocysteine	0	0	0	0	0	0	0	7	0	0
Histidine	0	2	0	4	0	2	7	0	0	0
Isoleucine	0	6	0	7	0	7	7	0	14	0
Leucine	0	4	0	8	0	5	7	0	14	0
Lysine	0	0	0	0	0	0	7	0	0	7
Methionine	0	3	0	1	0	3	7	0	0	0
Ornithine	4	4	1	10	6	4	7	0	0	14
Phenylalanine	10	0	10	0	8	3	7	0	0	14
Proline	4	1	4	0	8	0	7	0	0	11
Serine	8	0	8	0	8	0	7	0	0	14
Threonine	0	3	0	1	0	3	7	0	0	0
Tryptophan	2	4	3	8	0	7	7	0	12	0
Tyrosine	9	0	7	0	8	3	7	0	0	13
Valine	0	3	0	8	0	4	7	0	14	0
(2-Aminoethyl)phosphonate										
2-Dehydro-3-deoxy-D-gluconate										
L-2-hydroxyisocaproate										
(R)-3-(4-Hydroxyphenyl)lactate	0	0	0	0	0	0	0	3	3	0
4-Aminobutanoate										
Acetate	10	0	8	0	8	2	1	0	0	14
Acetaldehyde	3	0	0	0	0	0	0	0	0	3
N-Acetyl-D-glucosamine										
R Acetoin	6	0	2	1	0	1	0	1	0	6
S Acetoin										
2-Oxoglutarate	2	4	1	8	0	10	0	7	14	0
(R,R)-2,3-Butanediol										
(S,S)-2,3-Butanediol										
Citrate										
Ethanol										
Formaldehyde	0	6	0	5	0	8	0	0	0	0
Formate	0	0	0	0	8	0	0	0	0	8
D-Fructose										
Fumarate										
D-Glucose	11	0	12	0	12	0	7	0	14	0
D-Gluconate										
Glycerol	11	0	12	0	7	0	0	0	14	0

Glycerol 3-phosphate										
Glycolate	0	6	0	9	0	12	0	0	0	14
Imidazole lactate	0	0	0	0	0	0	0	4	4	0
D-Lactate										
L-Lactate										
Malate	0	3	0	0	0	2	0	0	3	0
Maltose										
Maltotetraose										
D-Mannose										
D-Mannitol										
Methylglyoxal										
Pyruvate	0	0	0	0	0	0	6	0	0	6
D-Sorbitol										
Succinate	0	4	0	2	0	0	0	1	6	0
Succinyl-CoA	6	3	3	4	0	11	0	0	14	0
Sucrose										
Trehalose										
Adenine	0	0	0	0	0	0	7	0	0	7
Adenosine										
Cytosine										
Cytidine										
Deoxyadenosine										
Deoxycytidine										
Deoxyribose										
dUMP	5	2	7	0	3	6	0	7	11	0
Deoxyuridine										
Guanine										
Hypoxanthine										
Inosine										
Orotate	4	0	3	0	0	0	0	1	1	5
Thymidine										
Uracil										
Uridine										
Xanthine										
Biotin (B7)	5	4	7	2	4	8	7	0	14	0
Coenzyme A	0	0	0	0	0	0	7	0	0	7
Dihydropteroate										
1-deoxy-D-xylulose 5-phosphate										
Folate (B9)	0	0	0	0	0	0	7	0	0	7
Nicotinate										
Nicotinamide D-ribonucleotide	2	3	2	3	0	11	7	0	14	0
Pyridoxine 5-phosphate (B6)	3	0	2	1	0	3	0	0	0	0
Pantothenate (B5)										
Pyridoxamine (B6)	0	0	0	0	0	0	1	0	1	0
Pyridoxal 5'-phosphate (B6)	0	0	0	0	0	0	3	2	13	0
Riboflavin (B2)	7	1	5	1	0	8	0	0	0	0
Tetrahydrofolate (B9)	4	1	3	0	0	5	0	0	0	0
Thiamin (B1)	0	0	0	0	0	0	7	0	0	7
Toxopyrimidine										
Ammonium	11	0	12	0	12	0	7	0	14	0
L-Cysteinylglycine										
L-methionyl-L-alanine										
Hydrogen sulfide	0	8	0	3	0	8	0	0	14	0
Sulfate	11	0	12	0	12	0	0	0	0	0

Table S5D. Effect of taxa, community size, and medium type on metabolite consumption and release rates. Tests with significant p values are shown in bold.

**Multivariate correlation**	Consumption		Production	
	Effect size (R^2)	P-value	Effect size (R^2)	P-value
AF	0.033	0.020	**0.061**	**0.002**
AP	0.026	0.030	0.027	0.046
AT	**0.155**	**0.001**	**0.047**	**0.006**
LB	**0.263**	**0.001**	**0.097**	**0.001**
LP	0.018	0.117	**0.066**	**0.001**
**PERMANOVA**	Effect test	Effect size (R^2)	Effect test	Effect size (R^2)
Taxon	**$F_{4,221} = 13.96$, $p = 0.001$**	**0.119**	**$F_{4,221} = 10.78$, $p = 0.001$**	**0.115**
Community size	$F_{4,221} = 0.918$, $p = 0.508$	0.008	$F_{4,221} = 2.04$, $p = 0.037$	0.022
Medium type	**$F_{2,221} = 90.67$, $p = 0.001$**	**0.387**	**$F_{2,221} = 43.56$, $p = 0.001$**	**0.232**
Community size * medium type	$F_{8,221} = 0.82$, $p = 0.681$	0.014	**$F_{8,221} = 2.10$, $p = 0.016$**	**0.045**
