# Supplementary material for: Predicted Metabolic Function of the Gut Microbiota of Drosophila melanogaster
Source: mSystems. 2021 May 4;6(3):e01369-20. doi: 10.1128/mSystems.01369-20 (PMC8269265; doi:10.1128/mSystems.01369-20)
Supplement: TABLE S6 [file msystems.01369-20-st006.pdf]

Table S6. Metabolites predicted to be available to the host.

Metabolite group	Metabolite	Nutrient replete	Base	Nutrient depleted
**Amino acid**	D-Alanine	■	■	■
	Homocysteine	■	■	■
	Methionine	■	□	□
	Ornithine	■	■	□
	Serine	■	□	□
**Central carbon**	Acetate	■	■	■
	2-Oxoglutarate	■	■	□
	Formaldehyde	□	□	■
	Formate	■	■	□
	Glycolate	□	■	■
	D-Lactate	■	□	□
	L-Lactate	■	□	□
	Malate	■	■	□
	Succinate	■	■	□
**Nitrogen**	Ammonium	■	■	□
**Nucleotide**	Hypoxanthine	■	□	□
	Uracil	■	□	□
	Xanthine	■	□	□
**Vitamin & cofactor**	CoA	■	□	□
	Pyridoxamine	■	■	□
	Thiamin	■	■	□
**Total number of metabolites**		19	12	5

Black rectangles indicate metabolite availability to host, grey rectangles indicate absence
