# Supplementary material for: Predicted Metabolic Function of the Gut Microbiota of Drosophila melanogaster
Source: mSystems. 2021 May 4;6(3):e01369-20. doi: 10.1128/mSystems.01369-20 (PMC8269265; doi:10.1128/mSystems.01369-20)
Supplement: TABLE S7 [file msystems.01369-20-st007.pdf]

Table S7A. List of components - rich medium.

Metabolite name	Exchange bound	Metabolite group
L-meso-2,6-Diaminoheptanedioate	-0.5	Amino acid
D-Alanine	-0.05	Amino acid
L-Alanine	-0.5	Amino acid
L-Arginine	-0.5	Amino acid
L-Asparagine	-0.5	Amino acid
L-Aspartate	-0.5	Amino acid
Choline	-0.05	Amino acid
Chorismate	-0.05	Amino acid
L-Cysteine	-0.5	Amino acid
L-Glutamine	-0.5	Amino acid
L-Glutamate	-0.5	Amino acid
Glycine	-0.5	Amino acid
Glycine betaine	-0.05	Amino acid
L-Homocysteine	-0.05	Amino acid
L-Histidine	-0.5	Amino acid
Histamine	-0.05	Amino acid
L-Isoleucine	-0.5	Amino acid
L-Leucine	-0.5	Amino acid
L-Lysine	-0.5	Amino acid
D-Methionine	-0.05	Amino acid
L-Methionine	-0.5	Amino acid
L-Methionine Sulfoxide	-0.05	Amino acid
Ornithine	-0.05	Amino acid
L-Phenylalanine	-0.5	Amino acid
L-Proline	-0.5	Amino acid
D-Serine	-0.5	Amino acid
L-Serine	-0.5	Amino acid
L-Threonine	-0.5	Amino acid
L-Tryptophan	-0.5	Amino acid
L-Tyrosine	-0.5	Amino acid
L-Valine	-0.5	Amino acid
Propane-1,2-diol	-0.05	Carbon
(2-Aminoethyl)phosphonate	-0.05	Carbon
2-Dehydro-3-deoxy-D-gluconate	-0.05	Carbon
L-2-hydroxyisocaproate	-0.05	Carbon
2-methyl butanoic acid	-0.05	Carbon
2-Methylbutanal	-0.05	Carbon
2-methylbutanol	-0.05	Carbon
2-methylpropanoic acid	-0.05	Carbon
2-methylpropanal	-0.05	Carbon
2-methylpropanol	-0.05	Carbon
(R)-3-(4-Hydroxyphenyl)lactate	-0.05	Carbon
3-methylbutanoic acid	-0.05	Carbon
3-methylbutanal	-0.05	Carbon
3-methylbutanol	-0.05	Carbon
4-Aminobutanoate	-0.05	Carbon
4-Aminobenzoate	-0.05	Carbon
Acetoacetyl-CoA	-0.05	Carbon
Acetate	-0.05	Carbon
Acetaldehyde	-0.05	Carbon
Acetol	-0.05	Carbon
N-Acetyl-D-galactosamine	-0.05	Carbon
N-Acetyl-D-glucosamine	-0.05	Carbon
(R)-Acetoin	-0.05	Carbon
(S)-Acetoin	-0.05	Carbon

2-Oxoglutarate	-0.05	Carbon
L-Arabinose	-0.05	Carbon
(R,R)-2,3-Butanediol	-0.05	Carbon
(S,S)-2,3-Butanediol	-0.05	Carbon
Benzaldehyde	-0.05	Carbon
cellobiose	-0.05	Carbon
Citrate	-0.05	Carbon
Dihydroxyacetone	-0.05	Carbon
Diacetyl	-0.05	Carbon
Ethanolamine	-0.05	Carbon
ethanol	-0.05	Carbon
formaldehyde	-0.05	Carbon
formate	-0.05	Carbon
D-Fructose	-0.05	Carbon
L-Fucose	-0.05	Carbon
Fumarate	-0.05	Carbon
D-Galactose	-0.05	Carbon
Galactitol	-0.05	Carbon
D-Glucose	-1	Carbon
D-Gluconate	-0.05	Carbon
Glycerol	-0.5	Carbon
Glycerol 3-phosphate	-0.05	Carbon
Glycolate	-0.05	Carbon
imidazole lactate	-0.05	Carbon
Indolelactate	-0.05	Carbon
D-Lactate	-0.05	Carbon
L-Lactate	-0.05	Carbon
Lactose	-0.05	Carbon
D-Malate	-0.05	Carbon
L-Malate	-0.05	Carbon
maltose	-0.05	Carbon
maltohexaose	-0.05	Carbon
maltopentaose	-0.05	Carbon
Maltotriose	-0.05	Carbon
maltotetraose	-0.05	Carbon
D-Mannose	-0.05	Carbon
Melibiose	-0.05	Carbon
Methional	-0.05	Carbon
D-Mannitol	-0.05	Carbon
Methylglyoxal	-0.05	Carbon
Orotate	-0.05	Carbon
Phenylacetaldehyde	-0.05	Carbon
Phenylethyl alcohol	-0.05	Carbon
Phenol	-0.05	Carbon
Phenyl lactate	-0.05	Carbon
Pyruvate	-0.05	Carbon
D-Ribose	-0.05	Carbon
D-Sorbitol	-0.05	Carbon
Succinate	-0.05	Carbon
Succinyl-CoA	-0.05	Carbon
sucrose	-0.05	Carbon
trehalose	-0.05	Carbon
D-Xylose	-0.05	Carbon
arsenite	-0.05	Inorganic ion
Calcium	-0.1	Inorganic ion
Cobinamide	-0.05	Inorganic ion
Cob(I)alamin	-0.05	Inorganic ion

Cadmium	-0.05	Inorganic ion
Chloride	-0.1	Inorganic ion
CO2	-0.05	Inorganic ion
Co2+	-0.1	Inorganic ion
Cu+	-0.05	Inorganic ion
Cu2+	-0.1	Inorganic ion
Fe2+	-0.1	Inorganic ion
Fe3+	-0.1	Inorganic ion
H+	-0.1	Inorganic ion
H2O	-1	Inorganic ion
Hg2+	-0.05	Inorganic ion
potassium	-0.1	Inorganic ion
Magnesium	-0.1	Inorganic ion
Mn2+	-0.1	Inorganic ion
Molybdate	-0.1	Inorganic ion
Sodium	-0.1	Inorganic ion
nickel	-0.1	Inorganic ion
o2	-2	Inorganic ion
Lead	-0.05	Inorganic ion
Phosphate	-0.1	Inorganic ion
Zinc	-0.1	Inorganic ion
Ammonium	-0.5	Nitrogen
Nitrite	-0.05	Nitrogen
Nitrate	-0.05	Nitrogen
Urea	-0.05	Nitrogen
5-Methylthio-D-ribose	-0.05	Nucleotide
Adenine	-0.05	Nucleotide
adenosine	-0.05	Nucleotide
Allantoin	-0.05	Nucleotide
Cytosine	-0.05	Nucleotide
cytidine	-0.05	Nucleotide
deoxyadenosine	-0.05	Nucleotide
deoxycytidine	-0.05	Nucleotide
Deoxyribose	-0.05	Nucleotide
deoxyuridine	-0.05	Nucleotide
Guanine	-0.05	Nucleotide
Hypoxanthine	-0.05	Nucleotide
inosine	-0.05	Nucleotide
thymidine	-0.05	Nucleotide
Uracil	-0.05	Nucleotide
uridine	-0.05	Nucleotide
Xanthine	-0.05	Nucleotide
L-alanyl-L-aspartate	-0.05	Peptide
L-alanyl-L-glutamine	-0.05	Peptide
L-alanyl-L-glutamate	-0.05	Peptide
L-alanylglycine	-0.05	Peptide
L-alanyl-L-histidine	-0.05	Peptide
L-alanyl-L-leucine	-0.05	Peptide
L-alanyl-L-threonine	-0.05	Peptide
Cys-Gly	-0.05	Peptide
Glycyl-L-asparagine	-0.05	Peptide
Glycyl-L-aspartate	-0.05	Peptide
Gly-Cys	-0.05	Peptide
Glycyl-L-glutamine	-0.05	Peptide
Glycyl-L-glutamate	-0.05	Peptide
Glycylleucine	-0.05	Peptide
Glycyl-L-methionine	-0.05	Peptide

Glycylphenylalanine	-0.05	Peptide
Glycylproline	-0.05	Peptide
Glycyl-L-tyrosine	-0.05	Peptide
L-methionyl-L-alanine	-0.05	Peptide
butanesulfonate	-0.05	Sulfur
ethanesulfonate	-0.05	Sulfur
Hydrogen sulfide	-0.5	Sulfur
Hexanesulfonate	-0.05	Sulfur
Isethionic acid	-0.05	Sulfur
L-Cysteate	-0.05	Sulfur
methanesulfonate	-0.05	Sulfur
Putrescine	-0.5	Sulfur
Sulfate	-0.5	Sulfur
Spermidine	-0.5	Sulfur
sulfoacetate	-0.05	Sulfur
Taurine	-0.05	Sulfur
Thiosulfate	-0.05	Sulfur
4-Amino-5-hydroxymethyl-2-methylpyrimidine	-0.1	Vitamin
5-Methyltetrahydrofolate	-0.1	Vitamin
Adenosylcobalamin	-0.05	Vitamin
Biotin	-0.1	Vitamin
CoA	-0.05	Vitamin
Dihydropteroate	-0.1	Vitamin
1-deoxy-D-xylulose 5-phosphate	-0.1	Vitamin
Folate	-0.05	Vitamin
Nicotinate	-0.1	Vitamin
Nicotinamide D-ribonucleotide	-0.05	Vitamin
Pyridoxine 5-phosphate	-0.05	Vitamin
Pantothenate	-0.1	Vitamin
Pyridoxamine	-0.1	Vitamin
Pyridoxal 5'-phosphate	-0.1	Vitamin
Pyridoxine	-0.1	Vitamin
riboflavin	-0.05	Vitamin
5,6,7,8-Tetrahydrofolate	-0.05	Vitamin
Thiamin	-0.05	Vitamin

Table S7B. List of components - base medium.

Metabolite name	Exchange bound	Metabolite group
L-meso-2,6-Diaminoheptanedioate	-0.05	Amino acid
L-Alanine	-0.05	Amino acid
L-Arginine	-0.05	Amino acid
L-Asparagine	-0.05	Amino acid
L-Aspartate	-0.05	Amino acid
L-Cysteine	-0.05	Amino acid
L-Glutamine	-0.05	Amino acid
L-Glutamate	-0.05	Amino acid
Glycine	-0.05	Amino acid
L-Histidine	-0.05	Amino acid
L-Isoleucine	-0.05	Amino acid
L-Leucine	-0.05	Amino acid
L-Lysine	-0.05	Amino acid
L-Methionine	-0.05	Amino acid
L-Phenylalanine	-0.05	Amino acid
L-Proline	-0.05	Amino acid
D-Serine	-0.05	Amino acid
L-Serine	-0.05	Amino acid

L-Threonine	-0.05	Amino acid
L-Tryptophan	-0.05	Amino acid
L-Tyrosine	-0.05	Amino acid
L-Valine	-0.05	Amino acid
D-Glucose	-0.1	Carbon
Glycerol	-0.05	Carbon
Calcium	-0.01	Inorganic ion
Chloride	-0.01	Inorganic ion
Co2+	-0.01	Inorganic ion
Cu2+	-0.01	Inorganic ion
Fe2+	-0.01	Inorganic ion
Fe3+	-0.01	Inorganic ion
H+	-0.01	Inorganic ion
H2O	-0.1	Inorganic ion
potassium	-0.01	Inorganic ion
Magnesium	-0.01	Inorganic ion
Mn2+	-0.01	Inorganic ion
Molybdate	-0.01	Inorganic ion
Sodium	-0.01	Inorganic ion
nickel	-0.01	Inorganic ion
o2	-2	Inorganic ion
Phosphate	-0.01	Inorganic ion
Zinc	-0.01	Inorganic ion
Ammonium	-0.05	Nitrogen
Putrescine	-0.05	Sulfur
Spermidine	-0.05	Sulfur
Hydrogen sulfide	-0.05	Sulfur
Sulfate	-0.05	Sulfur
4-Amino-5-hydroxymethyl-2-methylpyrimidine	-0.01	Vitamin
5-Methyltetrahydrofolate	-0.01	Vitamin
Biotin	-0.01	Vitamin
Dihydropteroate	-0.01	Vitamin
1-deoxy-D-xylulose 5-phosphate	-0.01	Vitamin
Nicotinate	-0.01	Vitamin
Pantothenate	-0.01	Vitamin
Pyridoxamine	-0.01	Vitamin
Pyridoxal 5'-phosphate	-0.01	Vitamin
Pyridoxine	-0.01	Vitamin

Table S7C. List of components - minimal medium.

Metabolite name	Exchange bound	Metabolite group
D-Glucose	-0.1	Carbon
Glycerol	-0.05	Carbon
Calcium	-0.01	Inorganic ion
Chloride	-0.01	Inorganic ion
Co2+	-0.01	Inorganic ion
Cu2+	-0.01	Inorganic ion
Fe2+	-0.01	Inorganic ion
Fe3+	-0.01	Inorganic ion
H+	-0.01	Inorganic ion
H2O	-0.1	Inorganic ion
potassium	-0.01	Inorganic ion
Magnesium	-0.01	Inorganic ion
Mn2+	-0.01	Inorganic ion
Molybdate	-0.01	Inorganic ion
Sodium	-0.01	Inorganic ion

nickel	-0.01	Inorganic ion
o2	-2	Inorganic ion
Phosphate	-0.01	Inorganic ion
Zinc	-0.01	Inorganic ion
Ammonium	-0.05	Nitrogen
Sulfate	-0.05	Sulfur
Pyridoxal 5'-phosphate	-0.01	Vitamin
